# Challenges associated with homologous directed repair using CRISPR-Cas9 and TALEN to edit the *DMD* genetic mutation in canine Duchenne muscular dystrophy

**DOI:** 10.1371/journal.pone.0228072

**Published:** 2020-01-21

**Authors:** Sara Mata López, Cynthia Balog-Alvarez, Stanislav Vitha, Amanda K. Bettis, Emily H. Canessa, Joe N. Kornegay, Peter P. Nghiem

**Affiliations:** 1 Department of Veterinary Integrative Biosciences, College of Veterinary Medicine and Biomedical Sciences, Texas A&M University, College Station, TX, United States of America; 2 Microscopy and Imaging Center, Texas A&M University, College Station, TX, United States of America; 3 Department of Pharmaceutical Sciences, School of Pharmacy and Pharmaceutical Sciences, Binghamton University, Johnson City, NY, United States of America; University of Houston, UNITED STATES

## Abstract

Duchenne muscular dystrophy (DMD) is caused by mutations in the *DMD* gene that abolish the expression of dystrophin protein. Dogs with the genetic homologue, golden retriever muscular dystrophy dog (GRMD), have a splice site mutation that leads to skipping of exon 7 and a stop codon in the *DMD* transcript. Gene editing via homology-directed repair (HDR) has been used in the mdx mouse model of DMD but not in GRMD. In this study, we used clustered regularly interspaced short palindromic repeats (CRISPR) and transcription activator-like effector nucleases (TALEN) to restore dystrophin expression via HDR in myoblasts/myotubes and later via intramuscular injection of GRMD dogs. In vitro, DNA and RNA were successfully corrected but dystrophin protein was not translated. With intramuscular injection of two different guide arms, sgRNA A and B, there was mRNA expression and Sanger sequencing confirmed inclusion of exon 7 for all treatments. On Western blot analysis, protein expression of up to 6% of normal levels was seen in two dogs injected with sgRNA B and up to 16% of normal in one dog treated with sgRNA A. TALEN did not restore any dystrophin expression. While there were no adverse effects, clear benefits were not seen on histopathologic analysis, immunofluorescence microscopy, and force measurements. Based on these results, methods must be modified to increase the efficiency of HDR-mediated gene repair and protein expression.

## Introduction

Duchenne muscular dystrophy (DMD) is a muscle wasting disease affecting 1 out of ~5,000 males worldwide. Mutations in the *DMD* gene eliminate expression of dystrophin protein [1]. Dystrophin deficiency leads to cycles of myofiber degeneration, necrosis and regeneration, with muscle eventually replaced by connective tissue and fat [2]. While there is currently no cure for DMD, recent treatment strategies have sought to restore the missing dystrophin protein by delivering part of the *DMD* gene [3] or by skipping the mutated area to restore the reading frame [4]. Successful *DMD* gene editing also has been achieved using clustered regularly interspaced short palindromic repeats (CRISPR) coupled with an enzyme, typically caspase (Cas)-9, in cell culture, the DMD murine model (mdx) [5–10], and, most recently, in the deltaE50-MD dog model for DMD [11]. Transcription activator-like effector nucleases (TALEN) has also been tested in vitro [7, 12, 13].

Most of these gene-editing studies have relied on non-homologous end joining (NHEJ) to ‘snip’ out mutated and out-of-frame exons [11–16], with apparent highly efficient correction in the deltaE50-MD dog [11]. Others have used homology-directed repair (HDR), typically relying on a donor clone to integrate with the targeted region to correct the *DMD* gene mutation [6–8, 10, 17]. Use of TALEN in vitro has shown even better performance [18]. To our knowledge, TALEN has not been used before in vivo, nor has CRISPR-HDR been used in large animals.

Gene editing with NHEJ seeks to produce a considerably milder Becker muscular dystrophy (BMD)-like phenotype associated with in-frame *DMD* gene mutations and must be employed early in the disease state for maximal benefit [11], In principle, compared to HDR, NHEJ achieves more successful gene editing but is also more prone to replicating errors [19]. An added advantage of HDR is that targeting mitotic cells in the S and G2 phases of the cell cycle alone may lead to efficient gene editing. New studies have emerged, showing a favorable outcome in post-mitotic cells, as well [20].

Golden retriever muscular dystrophy (GRMD) occurs naturally and is genetically homologous with DMD. Affected dogs largely recapitulate the progressive and variable phenotype observed in human patients [21]. A mutation in the *DMD* intron 6 acceptor splice site of GRMD dogs disrupts the reading frame, leading to exon 7 skipping, a stop codon in exon 8, and subsequent loss of dystrophin protein [22]. Both AAV-mediated gene replacement and exon skipping have been used in the GRMD model to produce a truncated dystrophin protein that could lead to a less severe phenotype typical of BMD [23]. Extending beyond these forms of gene therapy, some small DMD mutations may be amendable to HDR-mediated gene editing to excise and replace the mutated area with a normal sequence [24].

In this study, CRISPR/Cas9 and TALEN were paired with a donor clone to perform HDR-mediated gene editing to restore dystrophin protein in GRMD myoblasts. We then extended this work by injecting gene-editing plasmids intramuscularly in a total of six GRMD dogs. Our results show that HDR-mediated gene editing can safely correct a *DMD* mutation in a large animal model but also point to challenges that must be addressed to improve efficiency.

## Materials and methods

### Plasmids

Plasmid constructs were designed and generated by the investigators and Genecopoeia. SgRNA constructs contained the mCherry sequence to confirm clone insertion into the cell, CMV and T7 promoters, and ampicillin and neomycin resistance genes. TALEN left and right arm constructs contained CMV and T7 promoters and ampicillin resistance genes. Donor clone plasmid, from 5’ to 3’, contained the following: CMV, a T2A ribosomal skip site, eGFP sequence, ampicillin and neomycin resistance genes, and the corrected sequence of intron 6 and the acceptor splice site and part of exon 7 (S1 Table). The eGFP construct was located in intron 6.

Glycerol stocks were grown in *E*. *coli* with ampicillin and DNA was purified according to the manufacturer’s protocol (Endofree Plasmid Maxi Kit, Qiagen). Purified plasmid identity was confirmed via PCR (S2 Table) and restriction digest (EcoRI, SbfI, AflII for 1h at 37°C).

SgRNA A was targeted for the 5’ region located in intron 6 before the GRMD mutation “5’… CTCTTAAGGAATGATGGGCA…3’” with TGG as a PAM sequence. SgRNA B was targeted for the 3’ region located in exon 7 after the GRMD mutation “5’…GCAGTCAGCCACACAACGCC…3’”, CCA was the PAM sequence. SgRNA C differed from SgRNA A by 5 bp.

TALEN left arm was targeted for the 5’ region located in intron 6 before the GRMD mutation “5’… tcttgtgaaatattgtaa…3’”. Right arm was targeted for the 3’ region located in the splice acceptor site of intron 6 in the GRMD mutation “5’…TTATGTGTGTGTGTTTCG…3’”.

Recommended plasmid DNA and Endofectin concentrations were used. For HDR-CRISPR sgRNA treated cells, 2.5μg of DNA vector was first incubated in OPTI-MEM media (Life Technologies) in equal parts for 5 minutes (min) and subsequently mixed with the transfection agent, Endofectin (Genecopoeia) for 30 min. The DNA plasmid/Endofectin mix was then incubated with 5x10^5^ myoblast cells (previously stained with PAX7 and desmin for myoblast lineage confirmation) in a 6-well plate with Endofectin (Genecopoeia). For an 8-well plate, 233ng of total DNA vector/Endofectin mix was incubated in each well (1*10^5^ cells/well). Control GRMD cells were not treated with any type of DNA but incubated in OPTI-MEM for 70h.

### Animal studies

All dogs were used and cared for according to principles outlined in the National Research Council’s Guide for the Care and Use of Laboratory Animals. Procedures in this study were approved by the Texas A&M IACUC through protocols 2018–0182 (Standard Operating Procedures-Canine X-Linked Muscular Dystrophy) and 2017–0148 (Gene Editing in Duchenne Muscular Dystrophy).

Briefly, for the myoblast culture studies, dogs were premedicated (intramuscular acepromazine 0.02 mg/kg, butorphanol 0.4 mg/kg, and atropine 0.04mg/kg) and anesthesia was induced with sevoflurane via inhalation. The vastus lateralis muscle from a 4-year-old normal and a 3-year-old GRMD dog and the biceps femoris muscle from a 4.5-month old GRMD dog were then biopsied using an open surgical procedure to extract myoblasts for the CRISPR studies.

Dogs used in the HDR-gene editing in vivo studies were anesthetized using the above protocol at baseline and three months after treatment [25] and tibiotarsal joint force measurements (twitch and tetanic extension and flexion) and eccentric contraction decrement were performed as previously described [21, 26]. After these baseline measurements, 1x10^14^ vector copies in a total volume of ~ 6ml plasmid or saline were injected percutaneously using a grid pattern, 0.05 ml per site, into the three tibiotarsal flexor muscles of the cranial tibial compartment (cranial tibialis [CT], long digital extensor [LDE], and peroneus longus [PL]). We hypothesized that flexion force generated by HDR-Tx muscles would increase or deteriorate at a lesser rate if there were a treatment effect. Dogs were humanely euthanized by barbiturate overdose 3 months after HDR-Tx and necropsies were completed.

### Cell culture

Myoblast extraction was performed using a pre-plate technique [27]. Tissue was placed in a 15ml conical tube with PBS (Corning Cellgro) and 1% penicillin/streptomycin (Gibco, Life technologies). Muscle and PBS were placed in a petri dish, minced finely with sterile surgical blades, suctioned with a Pasteur pipette, and placed in 0.1% of collagenase (Roche Diagnostics) in DMEM (with Glutagro, Corning Cellgro). The cell lysate was incubated for 1h at 37°C with agitation at 120 rpm, digested every 15 min with a Pasteur pipette, washed with PBS twice, and re-suspended in 5ml of 0.05% trypsin (EDTA, Gibco, Life technologies) for 30 min at 37°C in a rocking incubator at 120 rpm. Trypsin was then inactivated with complete growth media (20% FBS -Pure grade VWR-, 2% chick embryo extract (USBiological), and 1% penicillin/streptomycin (Gibco, Life technologies). The sample was finally passed through two nylon strainers (100μm and 40μm) in a tube, plated in a collagen coated (Sigma) 6-well plate, and proliferated in growth media (20% FBS–VWR-, 2% chick embryo extract -Fisher scientific- 1% penicillin/streptomycin -Gibco, Life technologies- in DMEM -with Glutagro, Corning Cellgro-) until 95% confluency. Cells were stained for Pax7 (DSHB) and desmin (Sigma) to confirm myoblast identity. Myoblasts were treated for 70h with plasmid DNA with the concentrations mentioned above. Genomic DNA, total RNA, and protein were then extracted separately from individual experiments.

### Genomic DNA extraction

70h after initial incubation with plasmids, genomic DNA was extracted from the myoblasts following kit manufacturer protocol (QIAamp DNA blood mini kit, Qiagen).

### RNA extraction

Myoblasts were differentiated into myotubes with differentiation media (2% horse serum–VWR-, 1% penicillin/streptomycin -Gibco, Life Technologies- in DMEM -with Glutagro, Corning Cellgro-) for 2–3 weeks. Cells were considered myotubes after cell fusion, contained multiple nuclei, and had an elongated-shaped morphology. In addition, developmental myosin heavy chain (Leica) staining was performed after 21 days to confirm myotube lineage. Myotubes and 200mg of muscle from injected and normal dogs were resuspended in Tripure and total RNA was extracted following the manufacturer’s protocol (Roche Diagnostics). The RNA concentrations in the individual samples were measured using Nanodrop 2000 spectrophotometer and assessed for quality on a 2100 Bioanalyzer (Agilent Technologies). The RIN value ranged from 8.2–10. Reverse transcription was performed following the manufacturer’s protocol with oligo dT and Superscript II (Invitrogen). The reactions consisted of 100ng of total RNA in a 50μl reaction, ultra-pure H_2_O, oligo dt (2.5μl of 500ng/μl) and random hexamer (0.48μl of 1mM stock) heated to 65°C for 5 min and cooled to room temperature. Superscript II (2μl), 5X 1^st^ Strand buffer (10μl), 0.1M DTT (5μl), 10mM dNTPs (2.5μl) and a RNase block Ambion’s Superasin (1μl) were all heated to 37°C for 1h and terminated by heating at 90°C for 5 min for the cell extract. In the case of muscle extracted RNA, reverse transcription was performed following the manufacturer’s protocol with gene specific primer (S3 Table) and Superscript II (Invitrogen). The reactions consisted of 100ng of total RNA in a 20μl reaction, ultra-pure H_2_O, gene specific primer (2pmole) and dNTP mix (10mM) heated to 65°C for 5 min and cooled in ice. Superscript II (2μl), 5X 1^st^ Strand buffer (10μl), 0.1M DTT (5μl), 10mM dNTPs (2.5μl) and a RNase block Ambion’s Superasin (1μl) were all heated to 42°C for 2 min and after the final addition of SuperScript II the mix was incubated at 42°C for 1h and terminated by heating at 70°C for 15 min.

### Immunostaining

Myoblast identity was confirmed via Pax7 and desmin immunostaining [28]. Pax7 (Hybridoma Bank) and desmin (Sigma) were incubated at 1:100 each overnight (ON) at 4°C in 4% PFA fixed cells. Secondary antibodies Alexa 488 (Thermo Scientific) was incubated at 1:500 for 1h in the dark at room temperature. DAPI was incubated for 5min at 1:2000. Myotubes identity was confirmed with myosin heavy chain developmental staining (Leica) at 1:100.

Cells were plated and treated with HDR-CRISPR in 8 well slide chamber, differentiated for 18–21 days into myotubes and fixed with 1% PFA. Dystrophin (NCL-Dys1, NCL-Dys2 Leica Novacastra) was incubated at 1:100 and 1:50 respectively ON at 4°C. Secondary antibodies Alexa 647 (Jackson Immunolabs) were incubated at 1:500 for 1h at room temperature. DAPI (Invitrogen) was incubated for 5 minutes at 1:2000.

Tissue cross sections were cut in a cryostat at 6 nm of thickness as published previously [24]. Sections were mounted on a microscope slide, hydrated for 1h in PBS and fixed with acetone for 10 min. Blocked with 1% HS for 1h and incubated with dystrophin antibodies following the same procedure as above.

### PCR

Primers were designed to ensure plasmid identity of the clones (S2 Table). The PCR product was later restriction digested with AflII and SbfI (New England Biolabs), and electrophoresed in an agarose gel.

QRT-PCR was performed in triplicate reactions for Power Sybr Green Master Mix for the primers designed “in-house”. PCR assays were performed on a 7900HT Fast Real-Time PCR System (Applied Biosystems). The qRT-PCR reactions consisted of 10μl of Power Sybr Green, 300nM of each forward and reverse primers (2μl of 3μM stock), 5.5μl of PCR grade water, and 0.5μl of each reverse transcription reaction (cDNA) with a total of 20μl per well. The cycling parameters on the 7900HT machine were: 50C° for 2 min, 95°C for 10 min, and cycling 40 repeats of 95°C for 15 sec and 60°C for 1 min. Assay was performed with a dissociation curve added to validate primers. Primers used (S3 Table, outside primers, and S4 Table) were for *DMD* mRNA and *HPRT1* (control). Off-target, predicted sites were tested via PCR for each of the treatments (S5 and S6 Tables) following manufacturers protocol (OneTaq, New England Biolabs).

### Nested PCR

Primary PCR was performed using the outside primers (S3 Table) and TaKaRa Ex Taq Polymerase Kit under the following conditions: 94°C for 30 sec; 98°C for 10 sec, 52°C for 30 sec, 72°C for 1 min (30 times); and 75°C for 5 min. The desired band (540bp) of this reaction was excised from a 1% agarose gel and used for secondary PCR with inside forward and reverse primers designed. Secondary PCR was performed under the same conditions. Gel electrophoresis was used to determine the quality of PCR products. PCR reaction was submitted for Sanger sequencing with in-house primers.

### Sanger sequencing

For DNA genotyping of cells, PCR bands that were restriction digested by Sau96I (New England Biolabs) were cut, ligated (T.A. Cloning Kit Life Technologies, One Shot TOP10 Invitrogen), transfected and grown in *E*. *coli*. DNA extracted (QIAprep spin miniprep kit, Qiagen) was later sequenced and submitted to Eton and to the Institute for Plants Genomics & Biotechnology at Texas A&M, College Station, for Sanger sequencing with M13 forward primer. Ligated samples were independently sent to Eton without being digested by Sau96I in order to calculate HDR efficiency. CDNA product obtained from nested PCR was sent to Eton for sequencing with in-house primer (S1 Table). Between 5 to 6 colonies were sequenced for each clone, for sgRNA B one colony came up positive. For TALEN-Tx, 2 out of 8 colonies came up with the correctly modified sequence.

### Protein extraction and Western blot

Protein was extracted from three different parts of each of the cranial tibial compartment muscles (for statistical comparisons) and only once from the pre-treatment biopsied vastus lateralis/biceps femoris muscle with 10% SDS, 5% B-Mercaptoethanol, 75mM Tris-HCl pH 6.8 and 10mM EDTA. Samples were homogenized for 5 min at 4°C. The protein extract was incubated on ice with protein and phosphatase inhibitors (Halt Protease & Phosphatase Thermo Scientific) for 30 min and then centrifuged. Supernatant was quantified using a BCA protein quantification kit (Pierce Rapid Gold Thermo Scientific) and Nanodrop was used to measure the quantification curve. 7.5% SDS-PAGE gels (TGX Stain-Free Bio-Rad) were casted and ran for 30min at 300V, 70mA in 1X running buffer (1% SDS, 1.92 M glycine, 250mM Tris pH 8.3 for 10X) and transferred to a PVDF membrane on ice for 1:07h at 100V, 300mA in transfer buffer (0.1% SDS, 20%MeOH, 192mM Glycine, 25mM Tris). For statistical purposes, each muscle had three different protein aliquots obtained from medial, distal and proximal parts of the muscle. Each protein aliquot was run with baseline muscle sample, making the number of times of n = 3 for each muscle. For β-spectrin and dystrophin staining, membranes were blocked in 5% Milk TBST (TBS and 0.1% Tween-20) for 1h in a rocker, then antibodies (β-spectrin 1:2000, dystrophin concentration as above) were incubated at 4°C with rocking agitation. Desmin (1:10,000) was also used in some cases as a loading control. Those membranes were blocked in 5% BSA in TBS. Three TBST washes of 5 min each were performed. Secondary antibodies used were the same mentioned above in the immunofluorescence (IF) microscopy section at β-spectrin (1:5000), dystrophin (1:10,000), and desmin (1:10,000) and were incubated for 1h at room temperature while rocking. Three TBST washes were performed before a final 5 min TBS wash and exposure to a chemiluminiscent substance (SuperSignal West Pico Thermo Scientific) for 30 sec. Film was placed on top of the membrane in the dark room for as much time as needed in each case. All western blot membranes and films are provided in full without modification in S1 and S2 Figs.

### Imaging

Confocal microscopy was performed using an Olympus FV1000 inverted confocal microscope (Olympus America, Waltham, MA) equipped with an UPLSAPO 20x/0.85 and 20x/0.75 oil immersion objective. Scanning was performed with the confocal aperture corresponding to 1 Airy unit in sequential mode to minimize spectral overlap. Z-stacks were acquired in some cases with 1.52 um/slice steps. Excitation and emission wavelengths for individual channels were as follows: DAPI (Ex. 405 nm, Em 425–475 nm); GFP/Alexa488 (Ex. 488 nm, Em. 500-530nm); Cy3 (Ex. 543, Em. 565–615), Cy5/Alexa647 (Ex. 633, Em. > 650 nm).

### Cell counts

IF stained cells and necrotic fiber counts were calculated for every 100 cells counted performed in a blinded manner. Three images at 20X objective were analyzed per muscle per dog with an average of ~ 80 muscle cells in each image. Positive cells were considered those with dystrophin signal throughout the entire sarcolemmal membrane. Necrotic fibers were counted on H&E stained slides. A minimum of two to three images taken at 20X, with an average of 80 myofibers per image, were analyzed for all seven muscles from each dog, Features of necrosis extended from hyalinization whereby myofibers were round, swollen, and hypereosinophilic to different stages of fragmentation and phagocytosis.

### Liquid chromatography tandem mass spectrophotometry (LC-MS/MS)

One aliquot consisting of 100 μg of protein for each time point was loaded per lane into a TGX pre-cast 7.5% gel (Bio-Rad, Hercules, CA, USA) and separated for 40 minutes using 300V and 70mA. Gel was stained with Biosafe Coomassie blue (Bio-Rad, Hercules, CA, USA) for 1h. The gel was imaged and bands of interest were excised and processed for in-gel digestion by trypsin following previously published protocol [29]. The resulting peptides were extracted from the gel piece, vacuum dried, and then resuspended in 10 μl of 0.1% formic acid for LC-MS/MS analysis. An aliquot of 5μl peptide solutions were injected onto a Q Exactive^™^ HF-X Hybrid Quadrupole-Orbitrap^™^ Mass Spectrometer connected to a Dionex UltiMate 3000 RS UPLC system. A two-hour gradient was run using a mobile phase A of 0.1% formic acid in water, and a mobile phase B of 0.1% formic acid in 80% acetonitrile. An EASY-Spray^™^ LC Column (75 um diameter, 500 mm length, pore size 100 Å, particle size 2 μm; Thermo Scientific) column was first equilibrated with 98% mobile phase A and 2% mobile phase B for 10 minutes, then 5–35% B gradient for 90 minutes, 35‐100% B for 5 minutes, 100‐100% B for 5 minutes, 100–2% B for 1 minute and 2% B for 10 minutes. Data-dependent acquisition was performed at 60,000 resolution for a scan mass range of 300–1000 m/z, with one full MS scan followed by 20 MS/MS scans.

Peptide identification was performed using the Sequest algorithm in Proteome Discoverer 2.2. A *canis lupus familiaris* proteome from Uniprot (Proteome ID: UP000002254, last edit: May 16, 2019) containing 25,496 proteins was used for protein identification. Peptides were matched using oxidation, carbamidomethylation and acetylation dynamic modifications at a 10 ppm mass tolerance. To define the cellular function of detected proteins, the more extensive human (*homo sapiens*) database from Uniprot (v2017-07-05) was used containing 42,182 proteins. that contained 172,501 entries, 20,432 of them reviewed (Swiss-Prot) and 152,069 unreviewed (TrEMBL) (last edit: July 30, 2019).

For SILAC quantification, 50ug of dog tissue and 25ug of spiked-in SILAC labeled cell extract was used. The combined protein extract was run in a gel, followed by in-gel digestion procedures, as described above. For purposes of identifying muscle-specific protein, the top band of the gel was excised and processed. For analysis, only proteins that had a ratio of 10 or below with 3 or more peptides detected were reported. Peptides with a higher or outlier ratio were excluded.

## Results

### HDR-mediated guides

**Fig 1:**
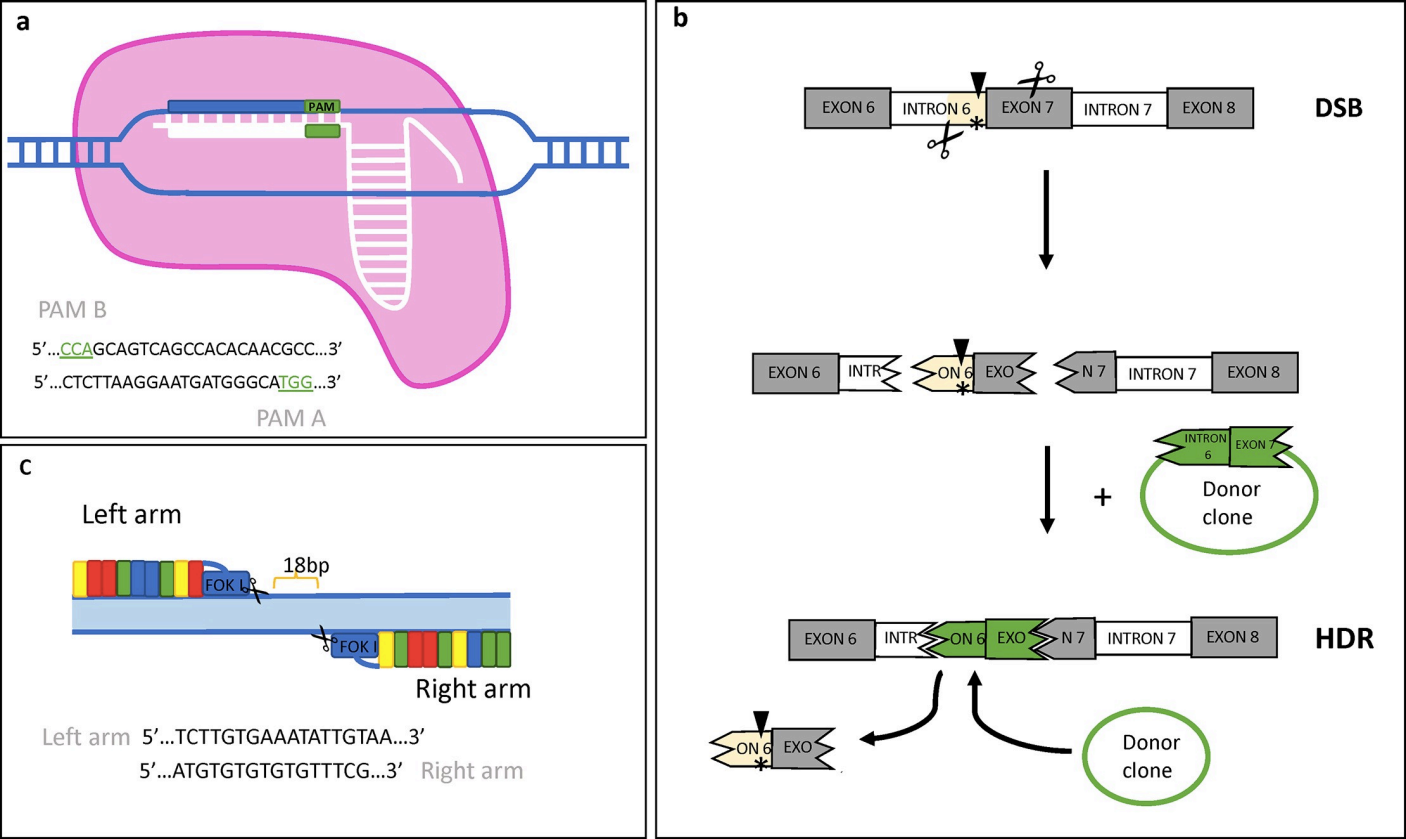
Experimental design of HDR-mediated CRISPR/Cas9 and TALEN gene editing for the GRMD mutation. **(a)** Guide selection included sgRNA A (PAM A underlined) and sgRNA B (PAM B underlined). **(b)** Experimental design. Double stranded breaks (DSB) occurred at the intron 6 area (highlighted) and/or at the exon 7 area to excise the GRMD mutation (asterisk). The donor clone (green) was used as a template for HDR to replace the excised area with the corrected *DMD* gene sequence. The black arrow designates the cutting site for Sau96I restriction enzyme, used to genotype GRMD dogs. When the dog does not have the mutated bp, Sau96I does not cut the DNA. **(c)** TALEN arm design with left and right sequences.

Canine myoblasts from GRMD dogs were treated (Tx) for 70 hours (h) with equal amounts of donor clone and either CRISPR/Cas-9 single guide RNA (sgRNA) plasmids (denoted as HDR-CRISPR) or TALEN left and right arms (denoted as HDR-TALEN; Fig 1). Left and right TALEN arms (Fig 1C) were incubated together; sgRNAs A and B, targeting different areas around the GRMD mutation (Fig 1A), were incubated with GRMD cells independently and together (sgRNA A&B). A donor clone was designed to include the correct *DMD* gene sequence at the intron 6 acceptor splice site-exon 7 boundary, enhanced green fluorescent protein gene (eGFP), and a cytomegalovirus (CMV) promoter (Fig 1B). An additional CRISPR guide with 5 mismatches to sgRNA A (sgRNA C) was used independently, combined with the donor clone and alone. A donor clone only treatment was also evaluated.

### HDR-treatment repairs the DNA at the myoblast level

**Fig 2:**
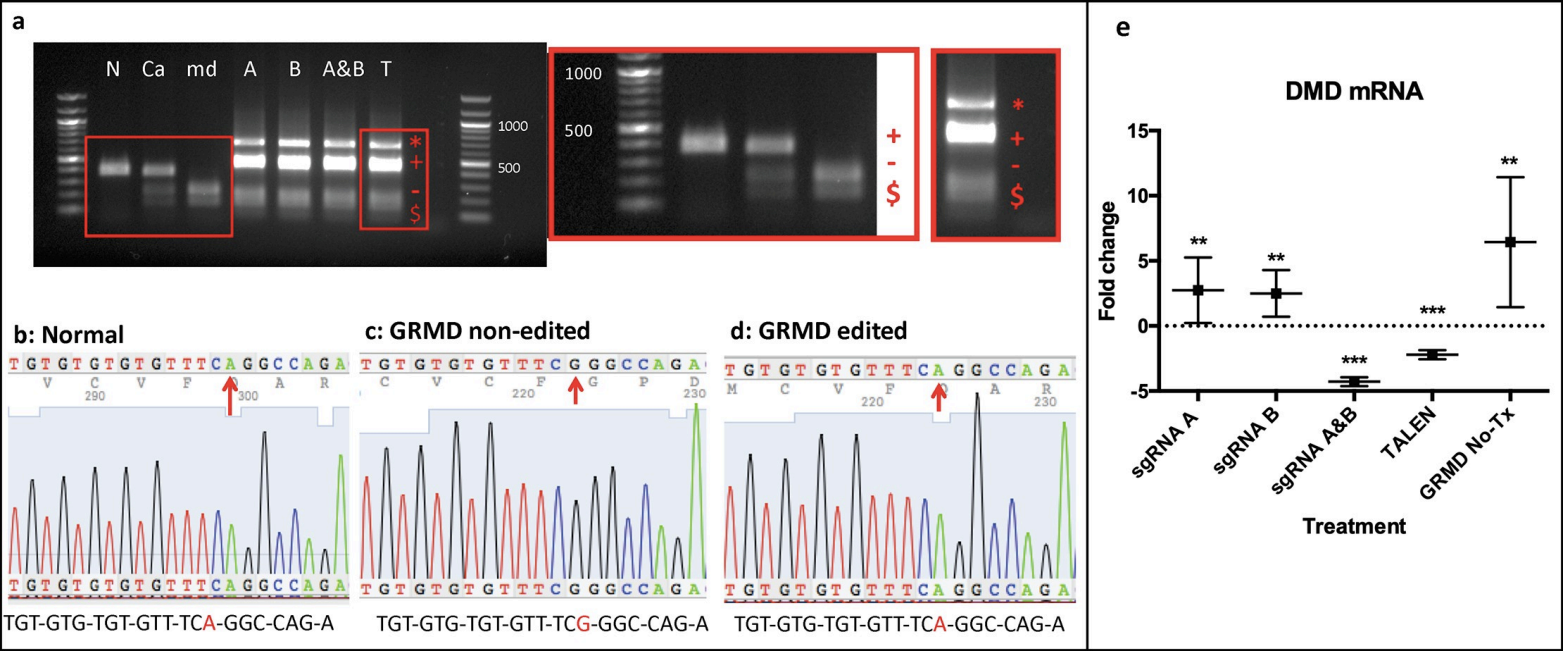
DNA and RNA analysis revealed HDR-mediated gene editing in GRMD-treated (Tx) myoblasts. **(a)** Agarose gel after PCR and restriction digest of GRMD-Tx and non-Tx cells. From left to right: Normal (N), carrier (Ca), non-Tx GRMD (md), GRMD-sgRNA A-Tx (A), GRMD-sgRNA B-Tx (B), GRMD-sgRNA A&B combined Tx (A&B), GRMD-TALEN Tx (T), ladder (100bp). All bands were sequenced: top band of ~ 700bp (red asterisk) matched part of the sequence of the donor clone after being cut with Sau96I enzyme, second band of ~ 500bp (red cross) was the corrected *DMD* gene sequence in GRMD-HDR-Tx samples and normal dog cells. This band was not cut with Sau96I. This second band had a higher molecular weight in GRMD-Tx cells compared to normal due to additional genes (eGFP) present in the donor clone. Third and fourth bands ~ 200bp (red dash, red cash sign) correspond to fragments of the GRMD mutated dog genome that was cut with Sau96I. **(b)** Sanger sequencing from the cut band (red cross) in a normal dog. Red arrow denotes the correct bp (A) in the *DMD* gene. **(c)** Sanger sequencing from the cut band in GRMD-HDR-Tx myoblasts but not successfully edited. Red arrow points at mutated bp (G) in GRMD dogs **(d)** Sanger sequencing from cut band in successfully GRMD-HDR-Tx GRMD cells. Red arrow denotes successfully replaced bp (A). **(e)** Dystrophin mRNA expression (mean±SE) among HDR treatments and normal cells compared to normal cells expression. *** p ≤ 0.001, ** p ≤ 0.01 vs Normal. Samples were analyzed using a pair wise fixed reallocation randomization test, excluding outliers with a Grubb’s test. Vertical bars indicate standard error of the mean. Treated myoblasts were differentiated into myotubes for 18 to 21 days and RNA was extracted from 6 replicates; values were normalized to *HPRT1* (house-keeping gene).

Genomic DNA from GRMD-HDR-treated (Tx) and non-Tx myoblasts was extracted and genotyped for the GRMD mutation [30] via Sau96I restriction digest (Fig 2). In this genotyping process, the PCR-amplified region was cut into two bands if the GRMD mutation was present; a normal dog would have one PCR band; and a carrier dog would have three bands (one normal band and one Sau96I digested band cut into two parts). GRMD-HDR-Tx cells showed one each of a mutated and wild-type *DMD* gene PCR band (Fig 2A), similar to the profile seen in carrier dogs [30], presumably due to less than 100% transfection efficiency. PCR bands of interest were cloned and Sanger sequencing confirmed inclusion of the corrected basepair (bp) in the splice site area of intron 6 (Fig 2B, 2C and 2D). TA cloning was performed to determine the efficiency of donor clone insertion into various clones: TALEN showed around 25% insertion efficiency and sgRNA B was approximately 16% effective, while other treatments did not yield positive colonies, presumably due to less than 10% efficacy, in line with levels seen in mdx mice treated in vivo [8, 10].

In some cases, Sanger sequencing in the region of interest showed that the wrong sequence of the donor clone had been inserted into the cell’s genome. Off-target, predicted sites within various genes were determined via webserver for CRISPR [31] and TALEN [32]. PCR analysis was performed for 9 (sgRNA A&B), 7 (sgRNA A) and 8 (sgRNA B) of the predicted genes. None showed banding differences between GRMD-HDR-CRISPR-Tx and GRMD non-Tx cells (S5 and S6 Tables) (S3 Fig). TALEN sequences did not match to any known genes for predicted off-target sites.

### HDR-treatment can modify mRNA at the myotube level

GRMD-HDR-CRISPR-Tx myoblasts were differentiated into myotubes for 18 to 21 days. Total RNA extraction and QRT-PCR for *HPRT1* and *DMD* genes were then quantified. *DMD* mRNA expression was compared to normal (dotted line Fig 2E, S7 Table) and was paradoxically increased in GRMD non-Tx cells versus normal. Using pair wise fixed reallocation randomization and Grubb’s outlier tests, with six replicates per treatment, *DMD* mRNA differed in all GRMD-HDR-Tx cells compared to normal except for sgRNA C combined with donor clone (S7 Table). SgRNA A&B and TALEN HDR-Tx showed decreased expression in the mRNA *DMD* transcript when compared to normal, while sgRNA A, sgRNA B, sgRNA C, donor clone alone and GRMD non-Tx cells increased their mRNA *DMD* expression. Once mRNA expression was compared between treatments (S7 Table), there were significant differences between sgRNA A with A&B, TALEN, sgRNA C and GRMD No-Tx; between sgRNA B and all the treatments except sgRNA A; and between sgRNA A&B and TALEN with all the treatments evaluated.

### HDR-treatment can modify protein at the myotube level

**Fig 3:**
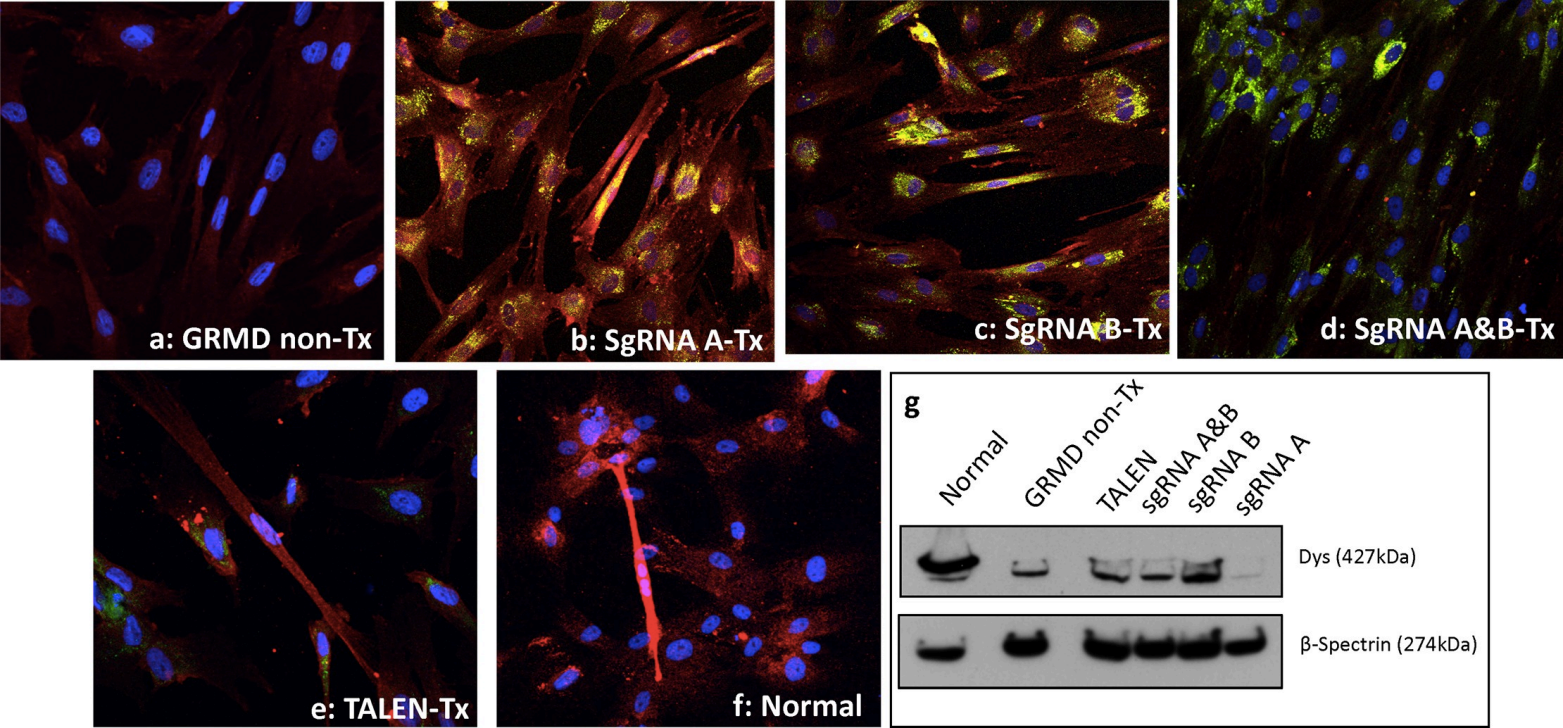
Dystrophin protein immunostaining and western blot from HDR- treated (Tx) and non-Tx myotubes. Dystrophin co-stained with C and N-terminus antibodies, Alexa 647 (red) and DAPI stained nuclei (blue). EGFP from the cells treated with donor clone (green) and mCherry from the sgRNA/Cas9 clones (yellow) indicate expression of plasmid proteins in the cell. All images were taken with the 20X objective; only multinucleated, elongated myotubes were used for quantification. **(a)** GRMD non-Tx myotubes showed background signal for Alexa 647. **(b)** sgRNA A-Tx and **(c)** B-Tx GRMD myotubes had increased dystrophin. **(d)** GRMD myotubes sgRNA A&B-Tx, **(e)** TALEN-Tx GRMD myotubes, and **(f)** normal myotubes. **(g)** Western blot from protein extracted from treated and non-Tx myotubes. Dystrophin co-stained with C and N-terminus antibodies and β-spectrin was used as a loading control.

In an independent experiment, GRMD-HDR-Tx myoblasts were differentiated into multi-nucleated, elongated myotubes and evaluated for dystrophin expression with IF (Fig 3A–3F) and western blotting (Fig 3G and S4 Fig). Non-Tx GRMD cells had significantly reduced dystrophin protein compared to normal cells (p < 0.05; S4A Fig). SgRNA A-Tx, sgRNA&B-Tx and TALEN-Tx cells showed partial dystrophin restoration (Fig 3 and S4A Fig), such that these values no longer differed statistically from normal. The values for neither SgRNA A-Tx nor sgRNA B-Tx differed from GRMD non-Tx cells, indicating no difference in the amount of dystrophin, and those treated with SgRNA A&B-Tx had lower levels of dystrophin (p < 0.01). TALEN-Tx cells showed the highest dystrophin expression in IF and differed statistically from GRMD non-Tx cells. Dystrophin expression for all HDR treatments was then compared to GRMD non-Tx cells via western blot, but no significant differences were observed after multiple replicates (Fig 3G and S4B and S4C Fig). Differences between both quantification methods are presumably due to the semi-quantitative nature of IF versus western blotting.

### Design of HDR-TX in GRMD dogs

Six GRMD dogs were treated in groups of two with each plasmid for HDR gene editing (sgRNA A, sgRNA B, TALEN; Table 1). A total of 1x10^14^ vector copies were injected percutaneously using a grid pattern into the three muscles (CT, LDE, and PL) of one cranial tibial compartment, while the other was injected with saline. Dogs were euthanized after 3 months and the muscles were removed for analysis. A biopsy from either the vastus lateralis or biceps femoris muscle was obtained before treatment to be used as a baseline for each dog.

**Table 1:** Study design for HDR-treated dogs. Dogs were assessed 3 months after treatment.

	Treatment	Dog	Age of injection	Sex
1	sgRNA A	Friendly	8yr	M
2	sgRNA A	Miercoles	5yr	F
3	sgRNA B	Bubbles	1.5yr	F
4	sgRNA B	Clove	4mos	F
5	TALEN	Hera	3mos	F
6	TALEN	Gantu	7mos	M

### HDR-TX alters DMD mRNA transcript in vivo

**Fig 4:**
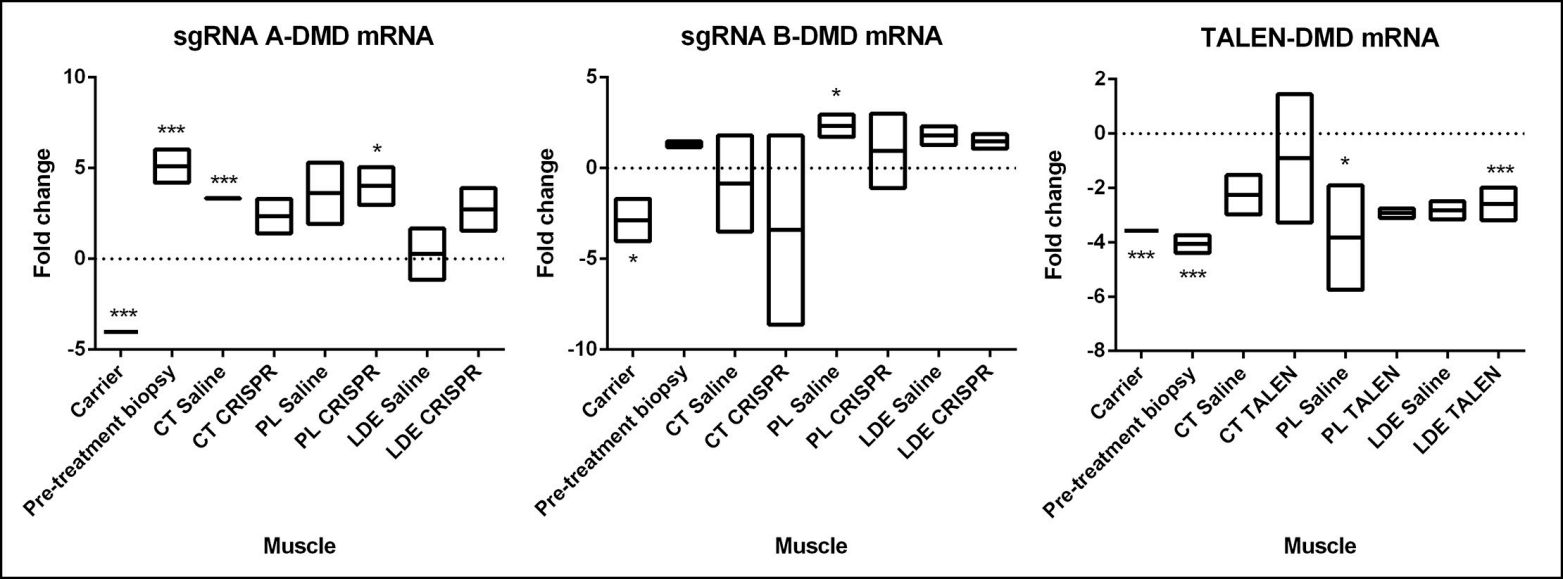
QRT-PCR results analyzed with a pair wise fixed reallocation randomization test. CT values for mRNA area between exon 6–8 of the *DMD* gene were normalized to *HPRT1* (house-keeping gene) as an internal control gene. Samples were analyzed using a pair wise fixed reallocation randomization test, excluding outliers with a Grubb’s test. Vertical boxes include standard error of the mean. RNA was extracted from each muscle and run in triplicate. All values were expressed as fold change compared to the normal dog values, which were also normalized to *HPRT1* (dotted line). CT = cranial tibial; PL = peroneus longus; LDE = long digital extensor; *p<0.05; ***p<0.001.

Quantification of the amount of transcript in the mutated area was performed via QRT-PCR (Fig 4) and confirmation of exon 7 insertion was observed via Sanger sequencing (S5 Fig). mRNA *DMD* expression in treated samples was averaged for each treatment group (n = 2 dogs for each HDR-Tx, with three replicates per muscle per dog) and compared to levels in normal (dotted line, Fig 4) and GRMD carrier dogs. SgRNA A treated dogs showed increased expression in *DMD* mRNA in their pre-treatment biopsy muscle compared to normal *DMD* mRNA levels, mirroring in vitro results. SgRNA A treated PL and saline treated CT had significantly increased *DMD* mRNA levels when compared to normal. No other muscles showed significant variability in the mRNA expression when compared to normal, potentially due to a systemic effect that caused normalization of *DMD* transcript after treatment (CT, LDE). When sgRNA B was used, the only significant differences were between the saline injected PL when compared to normal muscle levels, while no differences were observed between normal and GRMD pre-treatment biopsy or any other treated muscle. However, sgRNA B injected CT showed a promising range in *DMD* mRNA expression, indicating variability between animals, one of which could be mirroring carrier-like *DMD* mRNA expression levels. HDR-TALEN Tx dogs showed significant differences between normal *DMD* mRNA levels and pre-treatment biopsy, the saline injected PL and the TALEN injected LDE, all of which had a significantly decrease *DMD* mRNA fold change when compared to normal *DMD* mRNA levels. Expression levels in both the other treated and saline control muscles did not differ from normal, potentially due to a systemic effect. Paradoxically, pre-biopsy mRNA levels of TALEN treated dogs had a significantly reduced fold change when compared to normal. SgRNA A levels were significantly increased when compared to normal and sgRNA B levels did not differ from normal. This could be explained by the age of the dog at the time of the biopsy (Table 1), in that TALEN dogs were the youngest and sgRNA A the oldest. Expression of *DMD* mRNA at the area studied (exon 6–8) increases with aging in the GRMD dogs (unpublished data Mata and Nghiem).

### HDR-TX can increase protein in vivo

**Fig 5:**
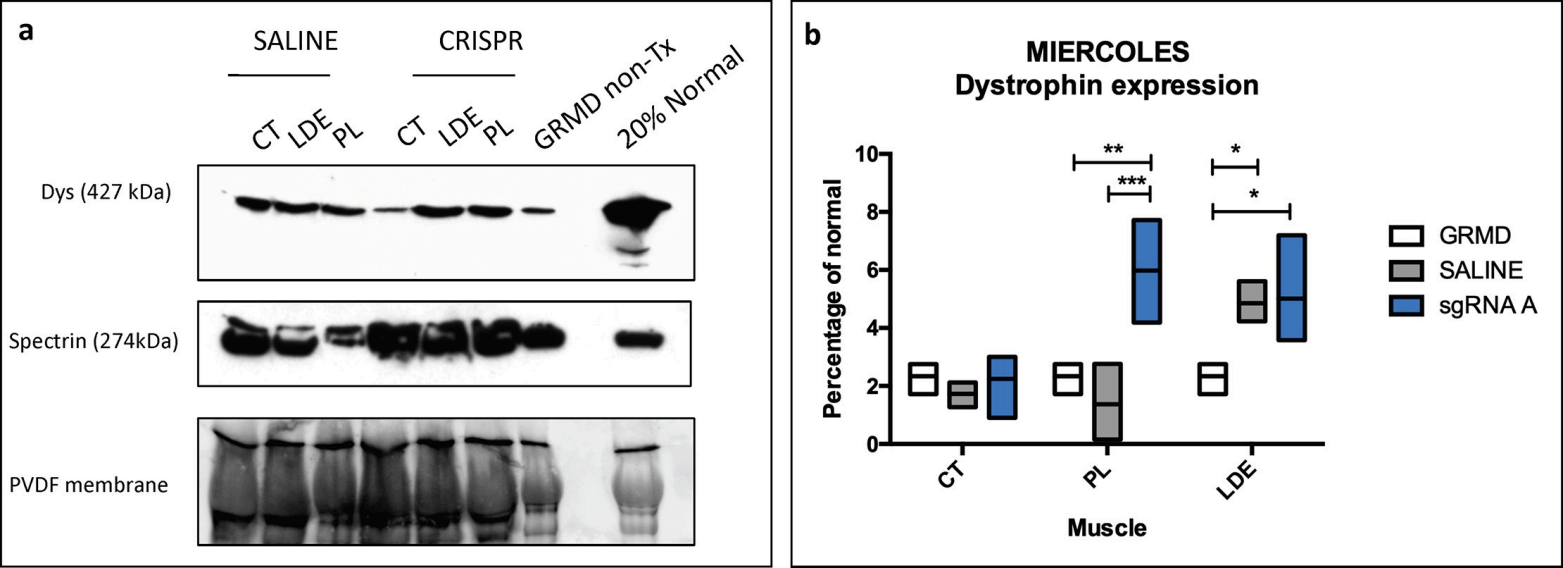
Dystrophin protein expression after HDR-CRISPR treatment in Miercoles. CRISPR sgRNA A was injected into one cranial tibial compartment and saline into the other. The vastus lateralis muscle from Miercoles (GRMD) was biopsied before injections to provide a general baseline and the HDR-CRISPR/saline muscles were harvested 3 months after treatment. **(a)** Dystrophin co-stained with C and N-terminus antibodies with goat anti-mouse secondary staining, β-spectrin stained as a muscle marker with goat anti rabbit secondary staining. Total protein from the PVDF membrane was used to normalize dystrophin. Normal sample was diluted to 20%. **(b**) Graph with dystrophin quantification for each muscle in the cranial tibial compartment. The PL and LDE muscles had increased dystrophin restoration in the HDR-CRISPR-Tx muscle compared to saline and GRMD while there were no differences in the HDR-CRISPR-Tx CT muscle. Statistical analysis was performed with Tukey’s multiple comparison’s test * p ≤ 0.05;** p ≤ 0.01;*** p ≤ 0.001. CT = cranial tibial; LDE = long digital extensor; PL = peroneus longus.

We then quantified dystrophin protein expression via western blot and IF. On western blot, only three (Bubbles, Miercoles, Friendly) of the six dogs showed dystrophin expression. Pre-treatment biopsy samples from the vastus lateralis and/or biceps femoris muscles had ~ 2–5% dystrophin levels compared to normal, consistent with the level of revertant fibers present in GRMD (Fig 5 and S6 and S7 Figs). Similar to the *DMD* transcript levels, the level of restored dystrophin expression in the GRMD-HDR-CRISPR injected limbs varied among the three dogs and treated muscles. Miercoles’ sgRNA A HDR-CRISPR-treated PL and LDE muscles had a modest increase of ~ 6% of normal levels (Fig 5) versus ~0–5% in the saline injected muscles (p < 0.001 for PL). Bubbles’ sgRNA B HDR-CRISPR-treated CT muscle showed the highest increase of approximately 16% of normal levels (S6 Fig). This was significantly higher than pre-treatment levels in her untreated vastus lateralis muscle. There were also 2–9% dystrophin levels in saline injected muscles, suggesting a possible systemic effect from the HDR-CRISPR treatment. The HDR-CRISPR- sgRNA A-Tx muscles in Friendly had dystrophin levels of ~2–5% of normal, consistent with the ~3–5% seen in untreated biceps femoris pre-treatment sample or saline-treated muscles. Levels in Friendly’s HDR-CRISPR-treated CT were lower than the saline-treated muscle (S7 Fig). Dystrophin protein was not detected in Clove, Gantu and Hera on multiple Western blots and the absence of expression was confirmed in CT samples analyzed via mass spectrometry with LC-MS/MS with SILAC (^13^C_6_ and ^15^N_2_ Lys and ^13^C_6_ Arg) labeled spike-in normal myoblasts (unpublished data). No dystrophin peptides were detected in any of the samples except normal dog control.

**Fig 6:**
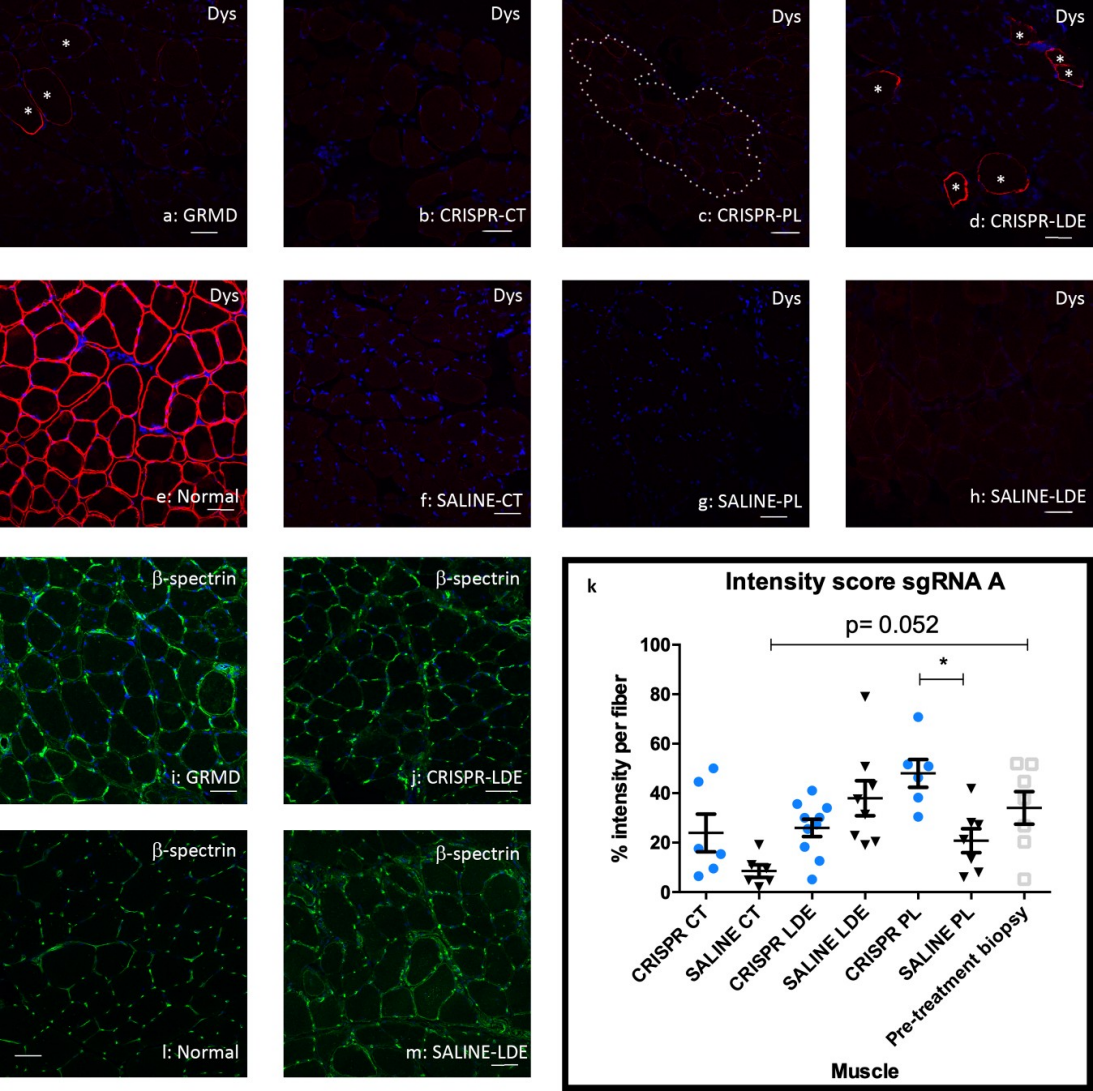
Dystrophin expression was minimally restored in HDR-CRISPR-Tx muscle from sgRNA A. Dystrophin co-stained with C and N-terminus antibodies with Alexa 647 (red), β -spectrin membrane control (green) and DAPI denotes the nuclei (blue). Asterisks denote cells with a value of 2 in intensity score for dystrophin signal in the GRMD non-Tx and Tx samples. Dotted line denotes cells with a value of 1 in intensity score. Scale bar = 50μm. **(a)** Pre-treatment biopsy sample for Miercoles. **(b)** HDR-CRISPR injected cranial tibial (CT) Miercoles. **(c)** HDR-CRISPR injected peroneus longus (PL) Miercoles. **(d)** HDR-CRISPR injected long digital extensor (LDE) Miercoles. **(e)** Normal dog muscle. **(f)** SALINE injected CT Miercoles. **(g)** SALINE injected PL Miercoles. **(h)** SALINE injected LDE Miercoles. **(i)** Pre-treatment biopsy sample for Miercoles. **(j)** HDR-CRISPR injected LDE Miercoles. **(k)** Dystrophin intensity quantification for Miercoles and Clove via One-way ANOVA multiple comparisons test, blue circle indicates CRISPR-Tx limb, black triangle indicates Saline-Tx limb, gray square indicates pre-treatment biopsied sample; *p<0.05. **(l)** Normal dog muscle **(m)** SALINE injected LDE Miercoles. Dys = dystrophin; p = p. value.

IF for dystrophin, β-spectrin and DAPI (Fig 6 and S8 and S9 Figs) were performed and quantified. Intensity was analyzed by providing a score of 0 to those fibers without signal, 1 to those fibers with partial signal and 2 to those fibers with high signal. The score was calculated by multiplying their signal (0, 1 or 2) by the number of fibers with such intensity. For the normal muscle 100% of the fibers have a score of 2, so the intensity score is 200%. Centrally nucleated fibers were also quantified. Values were averaged for the two dogs in each treatment group and the results were expressed as intensity score/central nuclei for every 100 muscle fibers. Notably, these data generally did not track with the Western blot results, perhaps reflecting the semi-quantitative nature of IF. B-spectrin was only used as a membrane control, but not normalized to dystrophin expression. Intensity score was evaluated per treatment, with sgRNA A showing differences between CRISPR and saline treated PL, and a decreasing trend in intensity between pre-treatment biopsy and saline CT (Fig 6). SgRNA B only showed significant differences between CRISPR treated LDE versus saline (S8 Fig). No other differences were observed for the HDR-CRISPR treated dogs. TALEN-HDR treated dogs showed an increase in intensity between CT and LDE TALEN treated muscles and their respective saline treated partners. An increase in intensity was also observed in CT and LDE when compared to the pre-treatment biopsied muscle (S9 Fig). Immunostaining results mirrored data obtained in the in vitro studies, with TALEN being the most effective treatment.

### HDR-Tx does not modify the amount of necrotic cells in tissue

**Fig 7:**
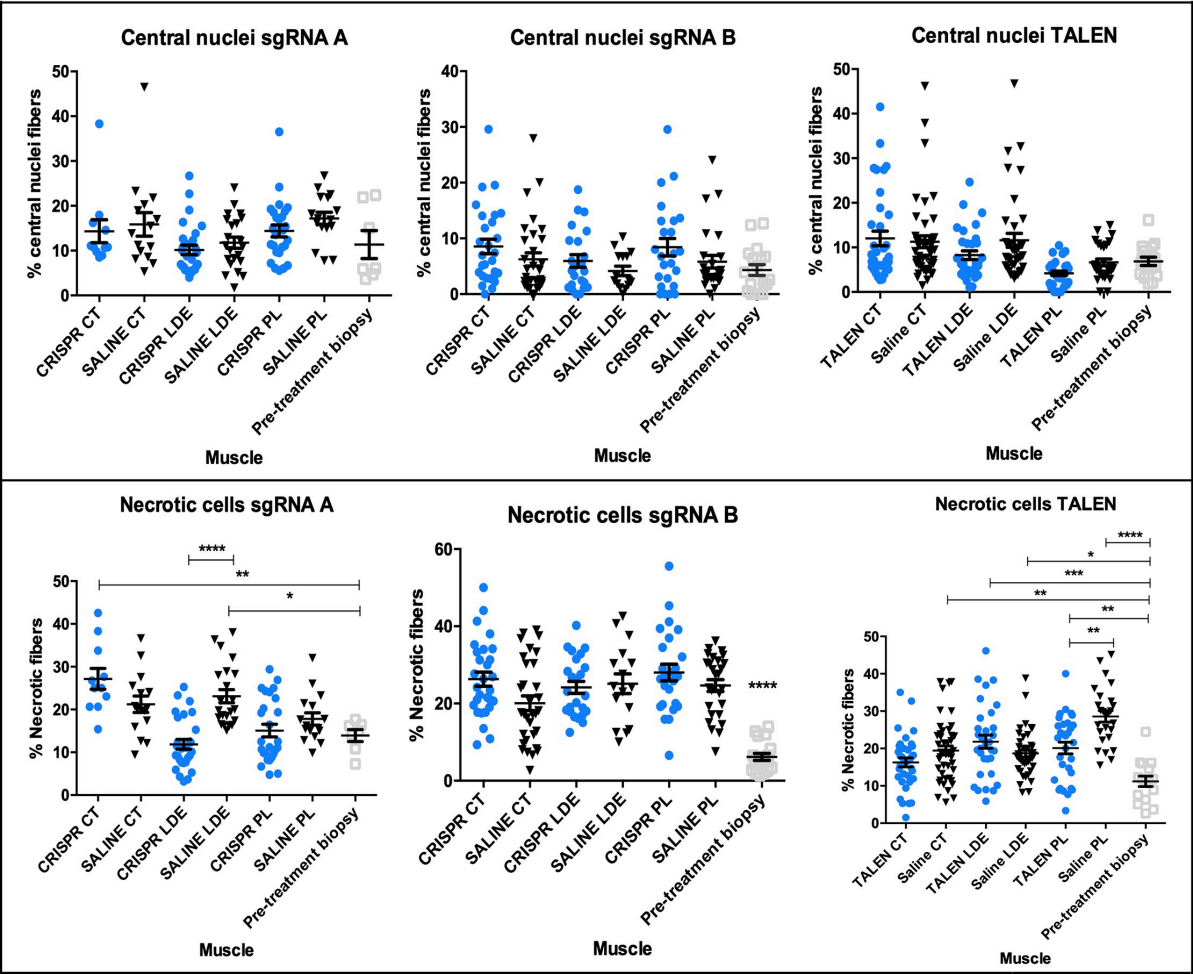
Percentage of central nuclei and necrosis from all three HDR treatments. Top: centrally nucleated fibers expressed as a percentage of fibers for every group treatment, sgRNA A, B and TALEN. Bottom: percentage of necrotic fibers for every treatment, sgRNA A, B (all significant versus pre-treatment biopsy) and TALEN. Blue circles denote HDR-injected limb while black triangle denotes saline-injected limb. Pre-treatment biopsy is indicated in grey square. Analysis was performed with One-way ANOVA multiple comparisons test. *p<0.05; **p< 0.01.; p***<0.001; p****<0.0001; CT = cranial tibial; LDE = long digital extensor; PL = peroneus longus.

Muscles were weighed at necropsy and necrotic fibers and centrally nucleated fibers were quantified for every hundred myofibers. Body-weight corrected muscle weight did not differ between treated and control muscles (S10 Fig). The number of central nuclei did not differ between baseline and treated samples (Fig 7). There were differences in the number of necrotic cells (Fig 7). For sgRNA A, the number of necrotic cells were increased in CRISPR CT and saline LDE compared individually to pre-treatment biopsy. Fewer necrotic fibers were seen in the CRISPR versus saline-treated LDE muscles. However, numbers did not differ from the pre-treatment values, possibly because any improvement was countered by natural disease progression. All muscles treated with SgRNA B had increased numbers of necrotic fibers compared to the pre-treatment samples. The same was true for all TALEN treated muscles, with the exception of one CT. The overall increase in necrotic fibers for all treatments when compared to biopsied pre-treatment samples could be due to the natural increase in necrosis and muscle wasting with GRMD disease progression. Values for muscle weight and dystrophin IF signal and between dystrophin IF signal and the number of necrotic fibers did not correlate (S11 Fig).

### HDR-tx does not improve functional measures in vivo

Force measurements and the degree of eccentric contraction decrement (ECD) were assessed before and after treatment. The mean degree of ECD showed no significant difference (p = 0.057) in the saline injected limbs post-treatment, as well as seen in those treated with CRISPR when compared to pre-treatment. Extension and flexion tibiotarsal tetanic force did not differ between treated and control limbs (S12 Fig). Cranial sartorius (CS) circumference measured during necropsy and compared between HDR-tx and Saline-tx limbs also did not differ (S8 Table) [21].

## Discussion

Expanding upon prior work in the mdx mouse and DMD cultured cells [6–8, 10, 17, 33], HDR-mediated gene editing with CRISPR/Cas-9 or TALEN to restore dystrophin expression was employed for the first time in the phenotypically relevant GRMD model of DMD. While up to 25% of editing was observed upon DNA analysis, these results did not track with protein levels, suggesting minimal to no rescue of HDR in treated dogs.

Starting with myoblasts in culture, the efficiency of donor clone insertion varied, with CRISPR sgRNA B being the most effective of its group at 16%, and TALEN at 25%. The other treatments had an efficiency of less than 10% in line with previously published HDR in vivo results from mdx mice [8, 10]. Somewhat unexpectedly, mRNA levels in GRMD myoblasts were higher than those of normal cells. Although dogma suggests that levels of *DMD* mRNA expression should be downregulated with frameshifting mutations due to nonsense-mediated decay, levels did not differ from wild type dogs in an earlier study [34]. Mdx values may be even higher than normal, especially with relatively 5’ mutations [35]. Importantly, mRNA expression is regulated by other factors such as microRNAs [36] and the presence of negative feedback loops [25]. Therefore, a reduction in transcript does not always correlate with protein expression. *DMD* mRNA levels in all myoblasts derived after HDR-treatment were lower than those from untreated GRMD cells and all were statistically significant compared to normal values, higher for sgRNA A and sgRNA B and lower for TALEN and sgRNA A&B. Values also differed between treatments, with sgRNA C combined with a donor clone coming closest to normal levels. However, values for neither donor clone nor sgRNA C alone did not differ when compared to GRMD no-Tx levels. At the protein level, GRMD-HDR-CRISPR-Tx myotubes had a modest restoration in dystrophin protein when compared to non-Tx GRMD cells. On the other hand, GRMD-HDR-TALEN-Tx cells did show a significant increase in protein expression. These CRISPR findings are in keeping with previously published data showing high efficiency on genome insertion that was not reflected at the protein level [10]. Consistent with the data for insertion efficiency, sgRNA B and TALEN were the most effective at restoring dystrophin protein. Based on these encouraging data, we performed in vivo studies.

In our standard preclinical studies, GRMD dogs are assessed between the ages of 3 and 6 months, corresponding roughly to 5–10 years in DMD, a period of relatively rapid deterioration in both diseases [23]. For sake of this preliminary in vivo study, we utilized six adult dogs ranging in age from 3 months to 8 years to establish proof-of principle of genetic correction using HDR. Given the late stage of disease for most dogs, phenotypic benefit was not necessarily expected. Our use of plasmids instead of adeno-associated viruses [37] (AAV) may have further limited the efficiency of genetic correction. Perhaps because of these limitations, while exon 7 in the *DMD* mRNA transcript was confirmed via Sanger sequencing, quantification of this area of the transcript via RT-QPCR did not fully track with the mRNA in vitro nor in vivo protein results. Of particular note, there was variability in the DMD mRNA content of the biopsies when compared to the same normal samples, indicating a potential relationship between transcript and age in the GRMD dog. Interestingly, these differences were not seen in culture, probably because of the presence of other regulators at the tissue level compared to single-cell culture [38]. HDR-TALEN provided the most encouraging results, with one of the treated limbs having decreased levels of mRNA compared to normal in both in vitro and in vivo studies. However, mRNA results did not track with protein. SgRNA A treated dogs showed dystrophin expression on Western blot. It was encouraging that PL CRISPR-Tx values tracked with dystrophin mRNA and Western blot in Miercoles, perhaps due to the small size of the muscle allowing a higher concentration of the injected plasmid. No dystrophin protein was detected in the TALEN treated dogs on Western blot or LC/MS-MS nor with Clove (sgRNA B-Tx), indicating a lack of HDR repair at the protein level.

Levels on Western blot and IF microscopy also did not track together, probably reflecting the respective quantitative versus semi-quantitative nature of these techniques [39]. Western data from the CT of Bubbles showed the highest correction of ~ 16% of normal. Dystrophin was only modestly increased, if at all, on Western analysis in other muscles from her and those of Friendly and Miercoles. Importantly, these modest levels of dystrophin expression are in keeping with the HDR gene-editing results in mdx mice [8, 10]. In another recently published canine CRISPR study, dystrophin expression was as high as 67% when compared to normal levels after AAV intramuscular CT injection using NHEJ [11]. Notably, HDR mediated gene editing is less efficient in post-mitotic cells compared to NHEJ [40, 41]. Previous studies have shown that a ~ 4% increase in dystrophin is necessary to lessen histopathologic lesions [42, 43]. Consistent with this finding, necrotic myofibers were not decreased in Miercoles’ LDE muscle compared to pre-treatment biopsy values. Not surprisingly, given the late stage of disease, there was no difference in force or ECD in treated and control limbs.

Results from both the CRISPR and TALEN treated dogs generally did not correspond to those of the in vitro studies. Miercoles was the only dog in which the sgRNA and protein values tended to track together. Thus, while the treatments appeared safe, improvements are needed in methodology before HDR can be applied to DMD. In future studies, better results might be achieved with a higher dose and a different system of delivery such as the other CRISPR dog study in which 2x10^13^ vg/kg was delivered intravenously [11], an earlier treatment window as mentioned above, a different type of vector delivery such as AAV [11, 44, 45], or improved techniques of HITI [46] or prime editing [47]. A combination of these improvements could lead to a higher percentage of HDR efficiency at both the DNA and protein levels.

## Supporting information

S1 Fig Full blots for the treated cells in culture. Western blots of the top part of the membrane after it was cut to stain for Dystrophin (Dys) and β-spectrin.
(TIFF)

S2 Fig Full blots and membranes for the treated dog muscles. Western blots of the top part of the membrane after it was cut to stain for Dystrophin (Dys), β-spectrin, desmin and PVDF membranes. **(a)** Miercoles **(b)** Friendly **(c)** Bubbles.
(TIFF)

S3 Fig Off target predicted genes analyzed with PCR. From left to right: 100bp ladder, sgRNA A&B DNA from six replicates 1–6, no-Tx GRMD DNA, negative control with PCR water. **(a)** EPG5 gene PCR with an expected band size of 505 bp. **(b)** PCBP3 gene PCR with an expected band size of 398bp. No differences were detected in either a or b.
(TIFF)

S4 Fig Dystrophin protein quantification in HDR-Tx cells. **(a)** Immunofluorescence microscopy: GRMD non-Tx cells had lower dystrophin expression compared to normal. Levels in sgRNA A-Tx, sgRNA B-Tx, sgRNA C, sgRNA C combined and donor clone only treated cells did not differ from normal, suggesting a potential treatment effect. However, this was not significantly different from non-Tx GRMD or normal cells. TALEN-Tx cells showed an increase in dystrophin protein when compared to non-Tx GRMD cells. Dystrophin expression for the two guides combined was significantly reduced compared to GRMD non-Tx and normal control. Intensity of dystrophin signal from multinucleated myotubes measured with ImageJ and analyzed via one way ANOVA. **** p ≤ 0.0001; * p ≤ 0.05. **(b)** Western blot: Dystrophin and β-spectrin signal for different treatments. β -spectrin was used as a loading control. **(c)** Western blot: Quantification of dystrophin signal normalized to normal myotubes protein extract. One way ANOVA was used and no statistical differences were found between treatments. Vertical bars indicate standard error of the mean.
(TIFF)

S5 Fig Sanger sequencing of DMD mRNA. Exon 7 boundary area sequenced from HDR-CRISPR-Tx muscle. Exon 7 was included in the *DMD* mRNA of the gene edited muscle.
(TIFF)

S6 Fig Western blot quantification of dystrophin expression from Bubbles, sgRNA B. **(a)** Dystrophin co-stained with C and N-terminus antibodies with goat anti mouse secondary staining, desmin stained as a muscle marker with the same secondary staining. PVDF membrane as reference. **(b)** Graph with dystrophin quantification for each muscle in the cranial tibial compartment. PVDF membrane was used as a loading control and values were normalized by comparing them to 20% of dystrophin protein extract from normal dog CT muscle. Triplicate blots for each sample were used as replicates. Statistical analysis was performed with Tukey’s multiple comparison’s test ** p ≤ 0.01; CT = cranial tibial; Dys = dystrophin; LDE = long digital extensor; PL = peroneus longus; Tx = treatment.
(TIFF)

S7 Fig Dystrophin protein expression after HDR-CRISPR treatment in Friendly. CRISPR sgRNA A was injected into one cranial tibial compartment and saline into the other. The biceps femoris (GRMD) was biopsied before injections and the HDR-CRISPR/saline muscles were harvested 3 months after treatment. **(a)** Dystrophin co-stained with C and N-terminus antibodies with goat anti mouse secondary staining, β-spectrin stained as a muscle marker. Total protein from the PVDF membrane was used to normalize dystrophin. Normal sample was diluted to 20%. **(b**) Graph with dystrophin quantification for each muscle in the cranial tibial compartment. Dystrophin in the saline-injected CT muscle was unexpectedly increased compared to the HDR-CRISPR-Tx CT muscle. Levels did not differ in the other HDR-CRISPR-Tx muscles. Statistical analysis was performed with Tukey’s multiple comparison’s test * p ≤ 0.05; CT = cranial tibial; LDE = long digital extensor; PL = peroneus longus.
(TIFF)

S8 Fig Dystrophin expression was minimally restored in HDR-CRISPR-Tx muscle from sgRNA B. Dystrophin co-stained with C and N-terminus antibodies with Alexa 647 (red), β -spectrin membrane control (green) and DAPI denotes the nuclei (blue). Asterisks denotes cells with a value of 2 in intensity score for dystrophin signal in the GRMD non-Tx and Tx samples. Scale bar = 50μm. **(a)** Pre-treatment biopsy sample for Bubbles **(b)** HDR-CRISPR injected cranial tibial (CT) Bubbles **(c)** HDR-CRISPR injected peroneus longus (PL) Bubbles **(d)** HDR-CRISPR injected long digital extensor (LDE) Bubbles **(e)** normal dog muscle **(f)** SALINE injected CT Bubbles **(g)** SALINE injected PL Bubbles **(h)** SALINE injected LDE Bubbles. **(i)** Pre-treatment biopsy sample for Bubbles **(j)** HDR-CRISPR injected CT Bubbles **(k)** dystrophin intensity quantification for Bubbles and Friendly via One-way ANOVA multiple comparisons test, blue circle indicates CRISPR-Tx limb, black triangle indicates Saline-Tx limb, gray square indicates pre-treatment biopsied sample; *p<0.05 **(l)** normal dog muscle **(m)** SALINE injected CT Bubbles. Dys = dystrophin.
(TIF)

S9 Fig Dystrophin expression was partially restored in HDR-TALEN-Tx muscle. Dystrophin co-stained with C and N-terminus antibodies with Alexa 647 (red), β -spectrin membrane control (green) and DAPI denotes the nuclei (blue). Asterisks denotes cells with a value of 2 in intensity score for dystrophin signal in the GRMD non-Tx and Tx samples, dotted line denotes cells with a value of 1 in intensity score. Scale bar = 100μm. **(a)** Pre-treatment biopsy sample for Hera **(b)** HDR-CRISPR injected cranial tibial (CT) Hera **(c)** HDR-CRISPR injected peroneus longus (PL) Hera **(d)** HDR-CRISPR injected long digital extensor (LDE) Hera **(e)** normal dog muscle **(f)** SALINE injected CT Hera **(g)** SALINE injected PL Hera **(h)** SALINE injected LDE Hera. **(i)** Pre-treatment biopsy sample for Hera **(j)** HDR-CRISPR injected LDE Hera **(k)** dystrophin intensity quantification for Hera and Gantu via One-way ANOVA multiple comparisons test, blue circle indicates CRISPR-Tx limb, black triangle indicates Saline-Tx limb, gray square indicates pre-treatment biopsied sample; **p<0.01; ***p<0.001; ****p<0.0001 **(l)** normal dog muscle **(m)** SALINE injected LDE Hera. Dys = dystrophin.
(TIF)

S10 Fig Muscle weights at necropsy normalized to body weight. Cranial tibialis muscle (CT), peroneus longus (PL) and long digital extensor (LDE) were cleaned and weighed at necropsy. Muscle weights were normalized to the body weight of the dog and analyzed via One-way ANOVA. No significant differences were detected. Blue square indicates HDR-Tx and gray circle indicates Saline. **(a)** HDR-CRISPR treated dogs **(b)** HDR-TALEN treated dogs.
(TIFF)

S11 Fig Correlations between dystrophin IF signal and muscle weight and necrotic fibers. Correlations were studied between dystrophin IF single and muscle weight at necropsy (top row) and dystrophin IF signal and percentage of necrotic fibers (bottom row). Blue circle indicates HDR treated limb, black circle indicates saline treated limb. No correlations were found in any of the treatments studied. From left to right: sgRNA A, sgRNA B, TALEN.
(TIFF)

S12 Fig Force normalized for body weight (N/kg) values from GRMD dogs before and after HDR treatment and eccentric contraction decrements (ECD). Circle is for pre-treatment values square is for post-treatment. **Top**: N = 4 analyzed via two way ANOVA. Blue color symbolizes sgRNA B’ data, grey color is for sgRNA A data. **Bottom:** N = 2 analyzed via two way ANOVA. **From left to right:** Extension tetanic values between saline and HDR- injected limbs as well as pretreatment and post-treatment. Flexion tetanic values. Eccentric contraction decrement (ECD). No statistical differences were found between saline and HDR-Tx limbs of GRMD dogs. In the saline injected limb for HDR-CRISPR ECD, there was a trend (p = 0.057) for an increase in ECD in post-treatment muscle compared to pre-treatment. The HDR-CRISPR injected limb ECD measurements were similar pre and post-treatment.
(TIFF)

S1 Table Donor clone plasmid entire sequence. (DOCX)

S2 Table Primers designed for clone purification and confirmation from bacterial stocks. (DOCX)

S3 Table Primers designed for nested PCR and gene specific primer to convert the DMD mRNA into cDNA. (DOCX)

S4 Table Primers designed for QRT-PCR after treatment with HDR. (DOCX)

S5 Table TOP 10 sgRNA A&B, TOP 8 sgRNA A and TOP 8 sgRNA B predicted off target effects. Capitalized letters indicate matching sequences to the predicted off target DNA location.
(DOCX)

S6 Table Primers designed for off target predicted genes. (DOCX)

S7 Table Dystrophin mRNA fold change. Treated myoblasts were differentiated into myotubes for 18 to 21 days and RNA was extracted from 6 replicates, values were normalized to *HPRT1* (house-keeping gene). Exons 28–29 were targeted for QRT-PCR. Fold change was calculated compared to the column treatment with the cells with gray backslash. F.change = fold change; SE = standard error; ***p≤ 0.001; ** p ≤ 0.01; * p ≤ 0.05. Samples were analyzed using a pair wise fixed reallocation randomization test, excluding outliers with a Grubb’s test. Sg RNA C combined denotes when SgRNA C was combined with donor clone.
(DOCX)

S8 Table Cranial Sartorius circumference variation expressed in millimeters/kg of body weight. (DOCX)

## References

[1] Hoffman EP, Brown RH Jr., Kunkel LM. Dystrophin: the protein product of the Duchenne muscular dystrophy locus. Cell. 1987;51(6):919–28. Epub 1987/12/24. 10.1016/0092-8674(87)90579-4 . 3319190

[2] Klingler W, Jurkat-Rott K, Lehmann-Horn F, Schleip R. The role of fibrosis in Duchenne muscular dystrophy. Acta Myol. 2012;31(3):184–95. Epub 2013/04/27. 23620650 PMC3631802

[3] Wang B, Li J, Xiao X. Adeno-associated virus vector carrying human minidystrophin genes effectively ameliorates muscular dystrophy in mdx mouse model. Proc Natl Acad Sci U S A. 2000;97(25):13714–9. Epub 2000/11/30. 10.1073/pnas.240335297 11095710 PMC17641

[4] Cirak S, Arechavala-Gomeza V, Guglieri M, Feng L, Torelli S, Anthony K, et al Exon skipping and dystrophin restoration in patients with Duchenne muscular dystrophy after systemic phosphorodiamidate morpholino oligomer treatment: an open-label, phase 2, dose-escalation study. Lancet. 2011;378(9791):595–605. Epub 2011/07/26. 10.1016/S0140-6736(11)60756-3 21784508 PMC3156980

[5] Long C, Amoasii L, Mireault AA, McAnally JR, Li H, Sanchez-Ortiz E, et al Postnatal genome editing partially restores dystrophin expression in a mouse model of muscular dystrophy. Science. 2016;351(6271):400–3. Epub 2016/01/02. 10.1126/science.aad5725 26721683 PMC4760628

[6] Long C, McAnally JR, Shelton JM, Mireault AA, Bassel-Duby R, Olson EN. Prevention of muscular dystrophy in mice by CRISPR/Cas9-mediated editing of germline DNA. Science. 2014;345(6201):1184–8. Epub 2014/08/16. 10.1126/science.1254445 25123483 PMC4398027

[7] Li HL, Fujimoto N, Sasakawa N, Shirai S, Ohkame T, Sakuma T, et al Precise correction of the dystrophin gene in duchenne muscular dystrophy patient induced pluripotent stem cells by TALEN and CRISPR-Cas9. Stem Cell Reports. 2015;4(1):143–54. 10.1016/j.stemcr.2014.10.013 25434822 PMC4297888

[8] Bengtsson NE, Hall JK, Odom GL, Phelps MP, Andrus CR, Hawkins RD, et al Corrigendum: Muscle-specific CRISPR/Cas9 dystrophin gene editing ameliorates pathophysiology in a mouse model for Duchenne muscular dystrophy. Nat Commun. 2017;8:16007 Epub 2017/06/24. 10.1038/ncomms16007 28643790 PMC5489999

[9] Nelson CE, Hakim CH, Ousterout DG, Thakore PI, Moreb EA, Castellanos Rivera RM, et al In vivo genome editing improves muscle function in a mouse model of Duchenne muscular dystrophy. Science. 2016;351(6271):403–7. 10.1126/science.aad5143 26721684 PMC4883596

[10] Lee Kunwoo, Conboy Michael, Hyo Min Park Fuguo Jiang, Hyun Jin Kim Mark A. Dewitt, et al Nanoparticle delivery of Cas9 ribonucleoprotein and donor DNA in vivo induces homology-directed DNA repair. Nature Biomedical Engineering. 2017;1(11):889–901. 10.1038/s41551-017-0137-2 29805845 PMC5968829

[11] Amoasii L, Hildyard JCW, Li H, Sanchez-Ortiz E, Mireault A, Caballero D, et al Gene editing restores dystrophin expression in a canine model of Duchenne muscular dystrophy. Science. 2018;362(6410):86–91. Epub 2018/09/01. 10.1126/science.aau1549 30166439 PMC6205228

[12] Ousterout DG, Perez-Pinera P, Thakore PI, Kabadi AM, Brown MT, Qin X, et al Reading frame correction by targeted genome editing restores dystrophin expression in cells from Duchenne muscular dystrophy patients. Mol Ther. 2013;21(9):1718–26. Epub 2013/06/05. 10.1038/mt.2013.111 23732986 PMC3776627

[13] Tang L, Bondareva A, Gonzalez R, Rodriguez-Sosa JR, Carlson DF, Webster D, et al TALEN-mediated gene targeting in porcine spermatogonia. Mol Reprod Dev. 2018;85(3):250–61. Epub 2018/02/03. 10.1002/mrd.22961 29393557 PMC6370346

[14] Tabebordbar M, Zhu K, Cheng JKW, Chew WL, Widrick JJ, Yan WX, et al In vivo gene editing in dystrophic mouse muscle and muscle stem cells. Science. 2016;351(6271):407–11. Epub 2016/01/02. 10.1126/science.aad5177 26721686 PMC4924477

[15] Yu HH, Zhao H, Qing YB, Pan WR, Jia BY, Zhao HY, et al Porcine Zygote Injection with Cas9/sgRNA Results in DMD-Modified Pig with Muscle Dystrophy. Int J Mol Sci. 2016;17(10). Epub 2016/10/14. 10.3390/ijms17101668 27735844 PMC5085701

[16] Ousterout DG, Kabadi AM, Thakore PI, Majoros WH, Reddy TE, Gersbach CA. Multiplex CRISPR/Cas9-based genome editing for correction of dystrophin mutations that cause Duchenne muscular dystrophy. Nat Commun. 2015;6:6244 Epub 2015/02/19. 10.1038/ncomms7244 25692716 PMC4335351

[17] Zhang Y, Long C, Li H, McAnally JR, Baskin KK, Shelton JM, et al CRISPR-Cpf1 correction of muscular dystrophy mutations in human cardiomyocytes and mice. Sci Adv. 2017;3(4):e1602814 Epub 2017/04/26. 10.1126/sciadv.1602814 28439558 PMC5389745

[18] He Z, Proudfoot C, Whitelaw CB, Lillico SG. Comparison of CRISPR/Cas9 and TALENs on editing an integrated EGFP gene in the genome of HEK293FT cells. Springerplus. 2016;5(1):814 Epub 2016/07/09. 10.1186/s40064-016-2536-3 27390654 PMC4916124

[19] Devkota S. The road less traveled: strategies to enhance the frequency of homology-directed repair (HDR) for increased efficiency of CRISPR/Cas-mediated transgenesis. BMB Rep. 2018;51(9):437–43. Epub 2018/08/15. 10.5483/BMBRep.2018.51.9.187 30103848 PMC6177507

[20] Nishiyama J, Mikuni T, Yasuda R. Virus-Mediated Genome Editing via Homology-Directed Repair in Mitotic and Postmitotic Cells in Mammalian Brain. Neuron. 2017;96(4):755–68 e5. Epub 2017/10/24. 10.1016/j.neuron.2017.10.004 29056297 PMC5691606

[21] Kornegay JN, Bogan JR, Bogan DJ, Childers MK, Li J, Nghiem P, et al Canine models of Duchenne muscular dystrophy and their use in therapeutic strategies. Mamm Genome. 2012;23(1–2):85–108. Epub 2012/01/06. 10.1007/s00335-011-9382-y 22218699 PMC3911884

[22] Sharp NJ, Kornegay JN, Van Camp SD, Herbstreith MH, Secore SL, Kettle S, et al An error in dystrophin mRNA processing in golden retriever muscular dystrophy, an animal homologue of Duchenne muscular dystrophy. Genomics. 1992;13(1):115–21. Epub 1992/05/01. 10.1016/0888-7543(92)90210-j . 1577476

[23] Kornegay JN. The golden retriever model of Duchenne muscular dystrophy. Skelet Muscle. 2017;7(1):9 Epub 2017/05/21. 10.1186/s13395-017-0124-z 28526070 PMC5438519

[24] Mata Lopez S, Hammond JJ, Rigsby MB, Balog-Alvarez CJ, Kornegay JN, Nghiem PP. A novel canine model for Duchenne muscular dystrophy (DMD): single nucleotide deletion in DMD gene exon 20. Skelet Muscle. 2018;8(1):16 Epub 2018/05/31. 10.1186/s13395-018-0162-1 29843823 PMC5975675

[25] Schneider SM, Sridhar V, Bettis AK, Heath-Barnett H, Balog-Alvarez CJ, Guo LJ, et al Glucose Metabolism as a Pre-clinical Biomarker for the Golden Retriever Model of Duchenne Muscular Dystrophy. Mol Imaging Biol. 2018 Epub 2018/03/07. 10.1007/s11307-018-1174-2 . 29508262 PMC6153676

[26] Kornegay JN, Spurney CF, Nghiem PP, Brinkmeyer-Langford CL, Hoffman EP, Nagaraju K. Pharmacologic management of Duchenne muscular dystrophy: target identification and preclinical trials. ILAR J. 2014;55(1):119–49. 10.1093/ilar/ilu011 24936034 PMC4158345

[27] Li Y, Pan H, Huard J. Isolating stem cells from soft musculoskeletal tissues. J Vis Exp. 2010;(41). Epub 2010/07/21. 10.3791/2011 20644509 PMC3156067

[28] Pawlikowski B, Lee L, Zuo J, Kramer RH. Analysis of human muscle stem cells reveals a differentiation-resistant progenitor cell population expressing Pax7 capable of self-renewal. Dev Dyn. 2009;238(1):138–49. Epub 2008/12/20. 10.1002/dvdy.21833 19097049 PMC2799339

[29] Jensen ON, Wilm M, Shevchenko A, Mann M. Sample preparation methods for mass spectrometric peptide mapping directly from 2-DE gels. Methods Mol Biol. 1999;112:513–30. Epub 1999/02/23. 10.1385/1-59259-584-7:513 . 10027274

[30] Bartlett RJ, Winand NJ, Secore SL, Singer JT, Fletcher S, Wilton S, et al Mutation segregation and rapid carrier detection of X-linked muscular dystrophy in dogs. Am J Vet Res. 1996;57(5):650–4. Epub 1996/05/01. . 8723876

[31] Bae S, Park J, Kim JS. Cas-OFFinder: a fast and versatile algorithm that searches for potential off-target sites of Cas9 RNA-guided endonucleases. Bioinformatics. 2014;30(10):1473–5. Epub 2014/01/28. 10.1093/bioinformatics/btu048 24463181 PMC4016707

[32] Doyle EL, Booher NJ, Standage DS, Voytas DF, Brendel VP, Vandyk JK, et al TAL Effector-Nucleotide Targeter (TALE-NT) 2.0: tools for TAL effector design and target prediction. Nucleic Acids Res. 2012;40(Web Server issue):W117–22. Epub 2012/06/14. 10.1093/nar/gks608 22693217 PMC3394250

[33] Doetschman T, Georgieva T. Gene Editing With CRISPR/Cas9 RNA-Directed Nuclease. Circ Res. 2017;120(5):876–94. Epub 2017/03/04. 10.1161/CIRCRESAHA.116.309727 . 28254804

[34] Cotten SW, Kornegay JN, Bogan DJ, Wadosky KM, Patterson C, Willis MS. Genetic myostatin decrease in the golden retriever muscular dystrophy model does not significantly affect the ubiquitin proteasome system despite enhancing the severity of disease. Am J Transl Res. 2013;6(1):43–53. Epub 2013/12/19. 24349620 PMC3853423

[35] Spitali P, van den Bergen JC, Verhaart IE, Wokke B, Janson AA, van den Eijnde R, et al DMD transcript imbalance determines dystrophin levels. FASEB J. 2013;27(12):4909–16. Epub 2013/08/27. 10.1096/fj.13-232025 . 23975932

[36] Cannell IG, Kong YW, Bushell M. How do microRNAs regulate gene expression? Biochem Soc Trans. 2008;36(Pt 6):1224–31. Epub 2008/11/22. 10.1042/BST0361224 . 19021530

[37] Lukashev AN, Zamyatnin AA Jr. Viral Vectors for Gene Therapy: Current State and Clinical Perspectives. Biochemistry (Mosc). 2016;81(7):700–8. Epub 2016/07/28. 10.1134/S0006297916070063 . 27449616

[38] Miki Y, Ono K, Hata S, Suzuki T, Kumamoto H, Sasano H. The advantages of co-culture over mono cell culture in simulating in vivo environment. J Steroid Biochem Mol Biol. 2012;131(3–5):68–75. Epub 2012/01/24. 10.1016/j.jsbmb.2011.12.004 . 22265957

[39] FDA. Peripheral and Central Nervous System Drugs Advisory Committee Meeting: FDA; 2016. https://www.fda.gov/downloads/AdvisoryCommittees/CommitteesMeetingMaterials/Drugs/PeripheralandCentralNervousSystemDrugsAdvisoryCommittee/UCM497063.pdf].

[40] Mao Z, Bozzella M, Seluanov A, Gorbunova V. Comparison of nonhomologous end joining and homologous recombination in human cells. DNA Repair (Amst). 2008;7(10):1765–71. Epub 2008/08/05. 10.1016/j.dnarep.2008.06.018 18675941 PMC2695993

[41] Li G, Zhang X, Zhong C, Mo J, Quan R, Yang J, et al Small molecules enhance CRISPR/Cas9-mediated homology-directed genome editing in primary cells. Sci Rep. 2017;7(1):8943 Epub 2017/08/23. 10.1038/s41598-017-09306-x 28827551 PMC5566437

[42] Li D, Yue Y, Duan D. Marginal level dystrophin expression improves clinical outcome in a strain of dystrophin/utrophin double knockout mice. PLoS One. 2010;5(12):e15286 Epub 2010/12/29. 10.1371/journal.pone.0015286 21187970 PMC3004926

[43] van Putten M, Hulsker M, Young C, Nadarajah VD, Heemskerk H, van der Weerd L, et al Low dystrophin levels increase survival and improve muscle pathology and function in dystrophin/utrophin double-knockout mice. FASEB J. 2013;27(6):2484–95. Epub 2013/03/06. 10.1096/fj.12-224170 23460734 PMC3659351

[44] Song Y, Morales L, Malik AS, Mead AF, Greer CD, Mitchell MA, et al Non-immunogenic utrophin gene therapy for the treatment of muscular dystrophy animal models. Nat Med. 2019 Epub 2019/10/09. 10.1038/s41591-019-0594-0 . 31591596 PMC7274039

[45] Nghiem PP, Kornegay JN. Gene therapies in canine models for Duchenne muscular dystrophy. Hum Genet. 2019;138(5):483–9. Epub 2019/02/09. 10.1007/s00439-019-01976-z . 30734120

[46] Suzuki K, Tsunekawa Y, Hernandez-Benitez R, Wu J, Zhu J, Kim EJ, et al In vivo genome editing via CRISPR/Cas9 mediated homology-independent targeted integration. Nature. 2016;540(7631):144–9. Epub 2016/11/17. 10.1038/nature20565 27851729 PMC5331785

[47] Anzalone AV, Randolph PB, Davis JR, Sousa AA, Koblan LW, Levy JM, et al Search-and-replace genome editing without double-strand breaks or donor DNA. Nature. 2019 Epub 2019/10/22. 10.1038/s41586-019-1711-4 . 31634902 PMC6907074

