# Correction: Challenges associated with homologous directed repair using CRISPR-Cas9 and TALEN to edit the *DMD* genetic mutation in canine Duchenne muscular dystrophy

**DOI:** 10.1371/journal.pone.0241430

**Published:** 2020-10-22

**Authors:** Sara Mata López, Cynthia Balog-Alvarez, Stanislav Vitha, Amanda K. Bettis, Emily H. Canessa, Joe N. Kornegay, Peter P. Nghiem

In the Abstract, there is an error in the seventh sentence. The terms sgRNA A and sgRNA B should be swapped. The correct sentence is: On western blot analysis, protein expression of up to 6% of normal levels was seen in two dogs injected with sgRNA A and up to 16% of normal in one dog treated with sgRNA B.

There is an error in the caption for Fig 6, “Dystrophin expression was minimally restored in HDR-CRISPR-Tx muscle from sgRNA A,” while referring to Miercoles and “Clove.” The correct caption should say Miercoles and “Friendly,” as per Table 1. Please see the complete, correct Fig 6 caption here.

**Fig 6:**
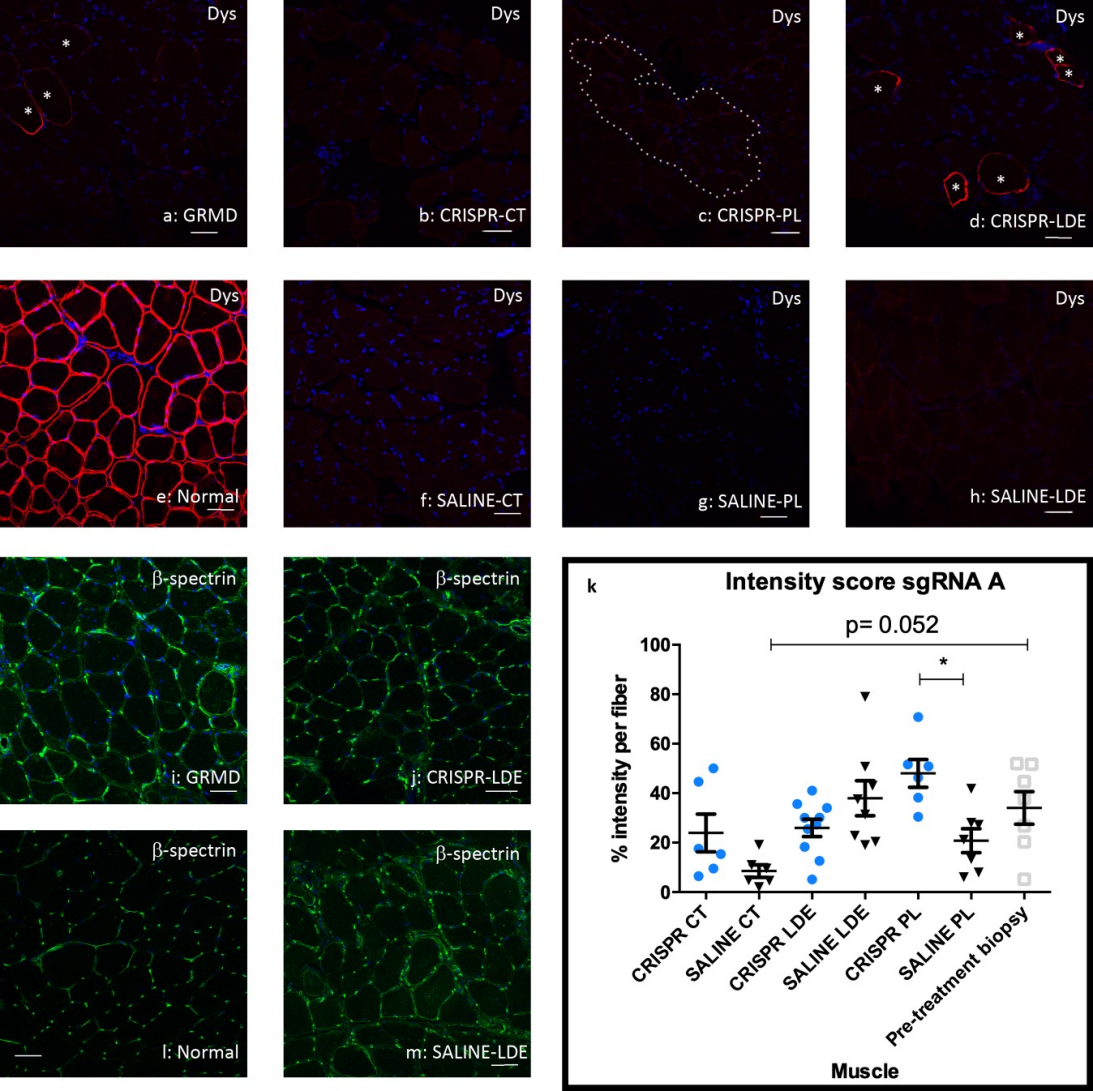
Dystrophin expression was minimally restored in HDR-CRISPR-Tx muscle from sgRNA A. Dystrophin co-stained with C and N-terminus antibodies with Alexa 647 (red), β -spectrin membrane control (green) and DAPI denotes the nuclei (blue). Asterisks denote cells with a value of 2 in intensity score for dystrophin signal in the GRMD non-Tx and Tx samples. Dotted line denotes cells with a value of 1 in intensity score. Scale bar = 50μm. **(a)** Pre-treatment biopsy sample for Miercoles. **(b)** HDR-CRISPR injected cranial tibial (CT) Miercoles. **(c)** HDR-CRISPR injected peroneus longus (PL) Miercoles. **(d)** HDR-CRISPR injected long digital extensor (LDE) Miercoles. **(e)** Normal dog muscle. **(f)** SALINE injected CT Miercoles. **(g)** SALINE injected PL Miercoles. **(h)** SALINE injected LDE Miercoles. **(i)** Pre-treatment biopsy sample for Miercoles. **(j)** HDR-CRISPR injected LDE Miercoles. **(k)** Dystrophin intensity quantification for Miercoles and Friendly via One-way ANOVA multiple comparisons test, blue circle indicates CRISPR-Tx limb, black triangle indicates SALINE-Tx limb, gray square indicates pre-treatment biopsied sample; *p<0.05. **(l)** Normal dog muscle **(m)** SALINE injected LDE Miercoles. Dys = dystrophin; p = p. value.

There is a similar error in the caption for S8 Fig, “Dystrophin expression was minimally restored in HDR-CRISPR-Tx muscle from sgRNA B,” while referring to Bubbles and “Friendly.” The correct caption should say Bubbles and “Clove,” as per Table 1. Please see the complete, correct S8 Fig here.

There is an error in the caption for S12 Fig, “Force normalized for body weight (N/kg) values from GRMD dogs before and after HDR treatment and eccentric contraction decrements (ECD).” The panel “Flexion tetanic values” should be cited before the panel “extension tetanic values.” Please see the complete, correct S12 Fig here.

In the Discussion, there is an error in the second sentence of the fifth paragraph. The correct sentence is: Miercoles was the only dog in which the mRNA and protein values tended to track together.

The images for S4 Fig and S5 Fig are incorrectly switched. The image that appears as S4 Fig should be S5 Fig, and the image that appears as S5 Fig should be S4 Fig. The figure captions appear in the correct order.

## Supporting information

S4 Fig Dystrophin protein quantification in HDR-Tx cells. **(a)** Immunofluorescence microscopy: GRMD non-Tx cells had lower dystrophin expression compared to normal. Levels in sgRNA A-Tx, sgRNA B-Tx, sgRNA C, sgRNA C combined and donor clone only treated cells did not differ from normal, suggesting a potential treatment effect. However, this was not significantly different from non-Tx GRMD or normal cells. TALEN-Tx cells showed an increase in dystrophin protein when compared to non-Tx GRMD cells. Dystrophin expression for the two guides combined was significantly reduced compared to GRMD non-Tx and normal control. Intensity of dystrophin signal from multinucleated myotubes measured with ImageJ and analyzed via one way ANOVA. **** p ≤ 0.0001; * p ≤ 0.05. **(b)** Western blot: Dystrophin and β-spectrin signal for different treatments. β -spectrin was used as a loading control. **(c)** Western blot: Quantification of dystrophin signal normalized to normal myotubes protein extract. One way ANOVA was used and no statistical differences were found between treatments. Vertical bars indicate standard error of the mean.
(TIFF)

S5 Fig Sanger sequencing of DMD mRNA. Exon 7 boundary area sequenced from HDR-CRISPR-Tx muscle. Exon 7 was included in the *DMD* mRNA of the gene edited muscle.
(TIFF)

S8 Fig Dystrophin expression was minimally restored in HDR-CRISPR-Tx muscle from sgRNA B. Dystrophin co-stained with C and N-terminus antibodies with Alexa 647 (red), β -spectrin membrane control (green) and DAPI denotes the nuclei (blue). Asterisks denotes cells with a value of 2 in intensity score for dystrophin signal in the GRMD non-Tx and Tx samples. Scale bar = 50μm. **(a)** Pre-treatment biopsy sample for Bubbles **(b)** HDR-CRISPR injected cranial tibial (CT) Bubbles **(c)** HDR-CRISPR injected peroneus longus (PL) Bubbles **(d)** HDR-CRISPR injected long digital extensor (LDE) Bubbles **(e)** normal dog muscle **(f)** SALINE injected CT Bubbles **(g)** SALINE injected PL Bubbles **(h)** SALINE injected LDE Bubbles. **(i)** Pre-treatment biopsy sample for Bubbles **(j)** HDR-CRISPR injected CT Bubbles **(k)** dystrophin intensity quantification for Bubbles and Clove via One-way ANOVA multiple comparisons test, blue circle indicates CRISPR-Tx limb, black triangle indicates Saline-Tx limb, gray square indicates pre-treatment biopsied sample; *p<0.05 **(l)** normal dog muscle **(m)** SALINE injected CT Bubbles. Dys = dystrophin.
(TIF)

S12 Fig Force normalized for body weight (N/kg) values from GRMD dogs before and after HDR treatment and eccentric contraction decrements (ECD). Circle is for pre-treatment values square is for post-treatment. **Top**: N = 4 analyzed via two way ANOVA. Blue color symbolizes sgRNA B’ data, grey color is for sgRNA A data. **Bottom**: N = 2 analyzed via two way ANOVA. **From left to right**: Flexion tetanic values. Extension tetanic values between saline and HDR-injected limbs as well as pretreatment and post-treatment. Eccentric contraction decrement (ECD). No statistical differences were found between saline and HDR-Tx limbs of GRMD dogs. In the saline injected limb for HDR-CRISPR ECD, there was a trend (p = 0.057) for an increase in ECD in post-treatment muscle compared to pre-treatment. The HDR-CRISPR injected limb ECD measurements were similar pre and post-treatment.
(TIFF)
