# Supplementary figures and images for: Challenges associated with homologous directed repair using CRISPR-Cas9 and TALEN to edit the DMD genetic mutation in canine Duchenne muscular dystrophy
Source: PLoS One. 2020 Jan 21;15(1):e0228072. doi: 10.1371/journal.pone.0228072 (PMC6974172; doi:10.1371/journal.pone.0228072)

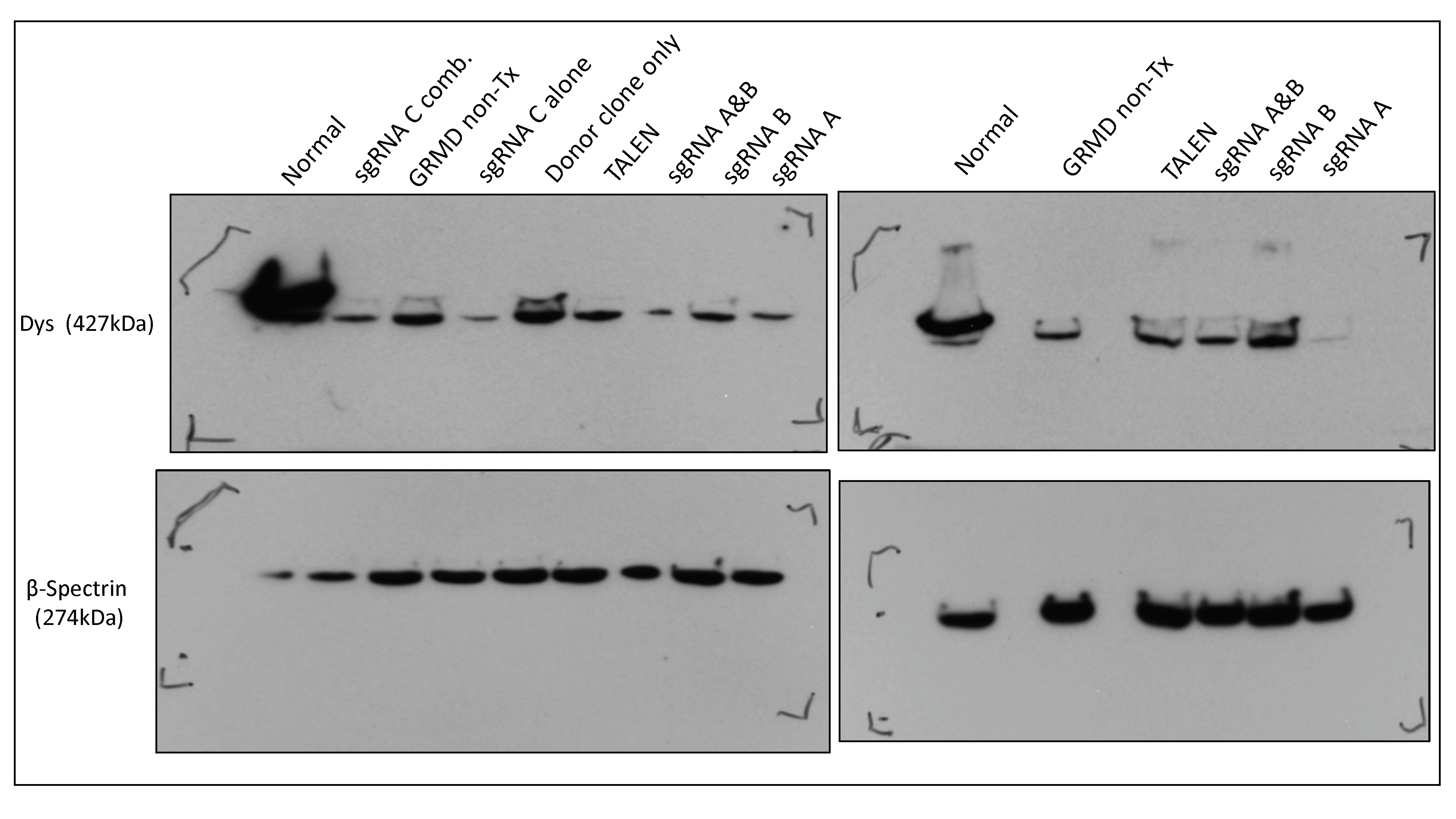

Supplement: S1 Fig — Western blots of the top part of the membrane after it was cut to stain for Dystrophin (Dys) and β-spectrin. (TIFF) [file pone.0228072.s001.tiff]

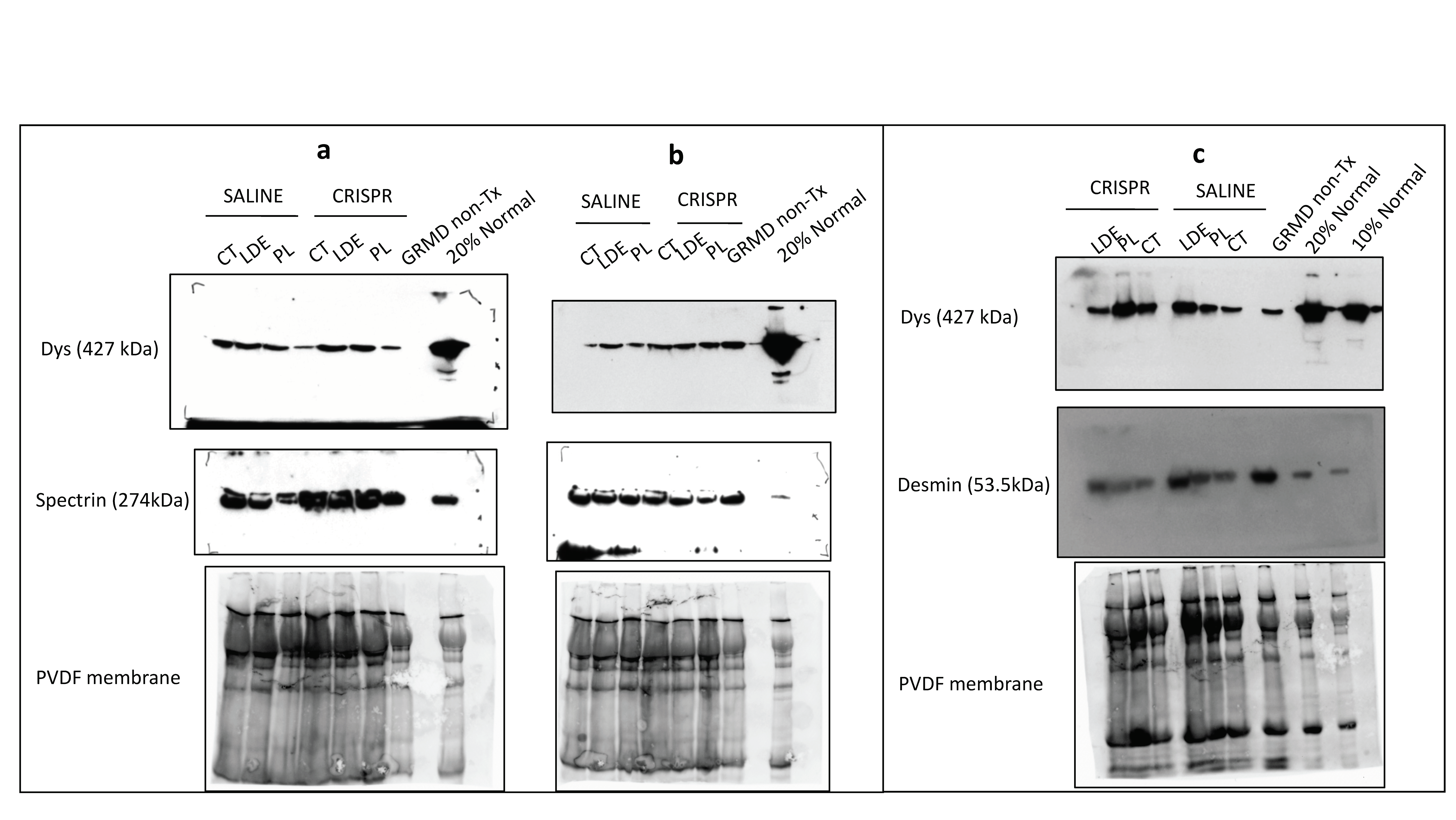

Supplement: S2 Fig — Western blots of the top part of the membrane after it was cut to stain for Dystrophin (Dys), β-spectrin, desmin and PVDF membranes. (a) Miercoles (b) Friendly (c) Bubbles. (TIFF) [file pone.0228072.s002.tiff]

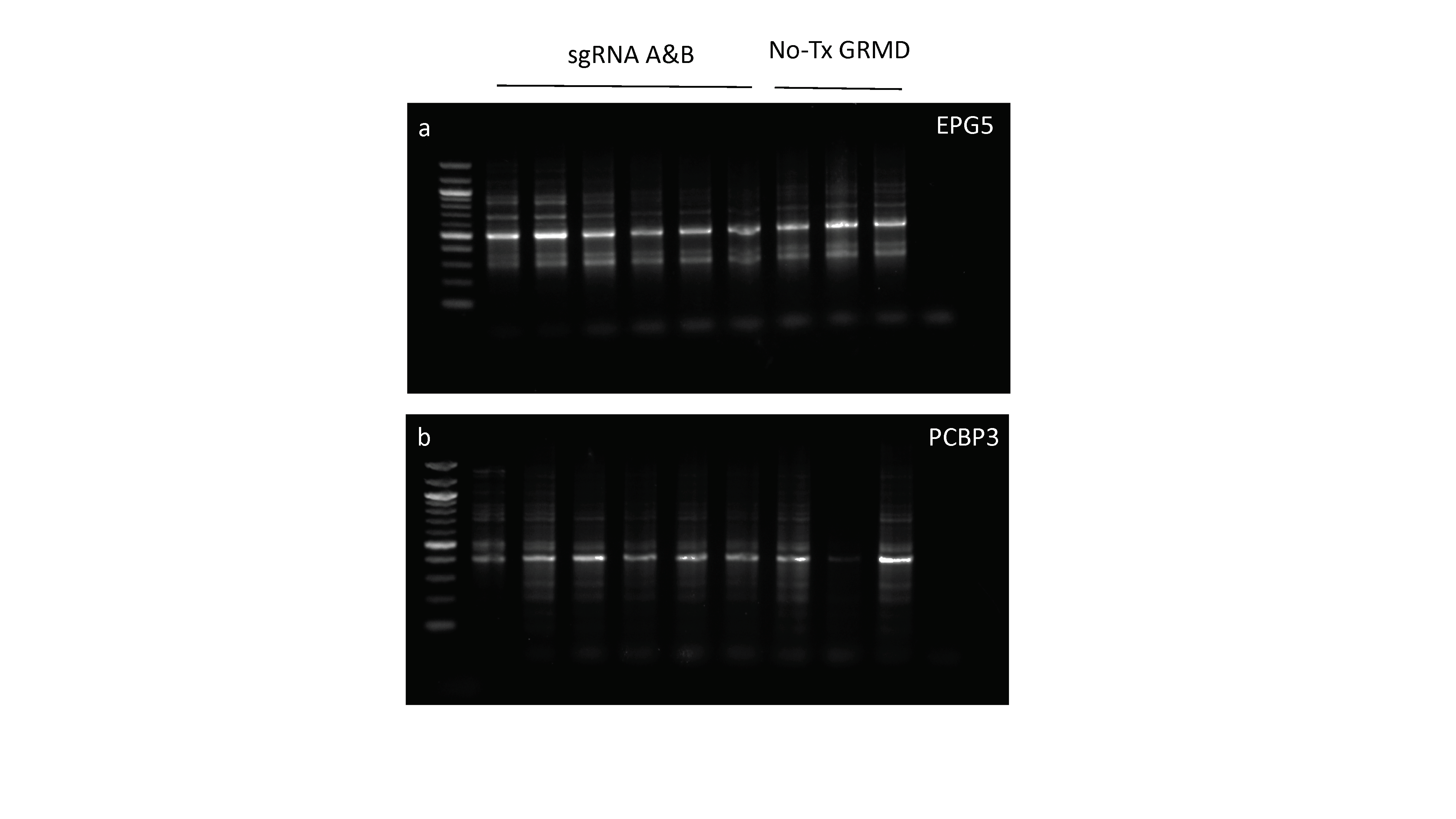

Supplement: S3 Fig — From left to right: 100bp ladder, sgRNA A&B DNA from six replicates 1–6, no-Tx GRMD DNA, negative control with PCR water. (a) EPG5 gene PCR with an expected band size of 505 bp. (b) PCBP3 gene PCR with an expected band size of 398bp. No differences were detected in either a or b. (TIFF) [file pone.0228072.s003.tiff]

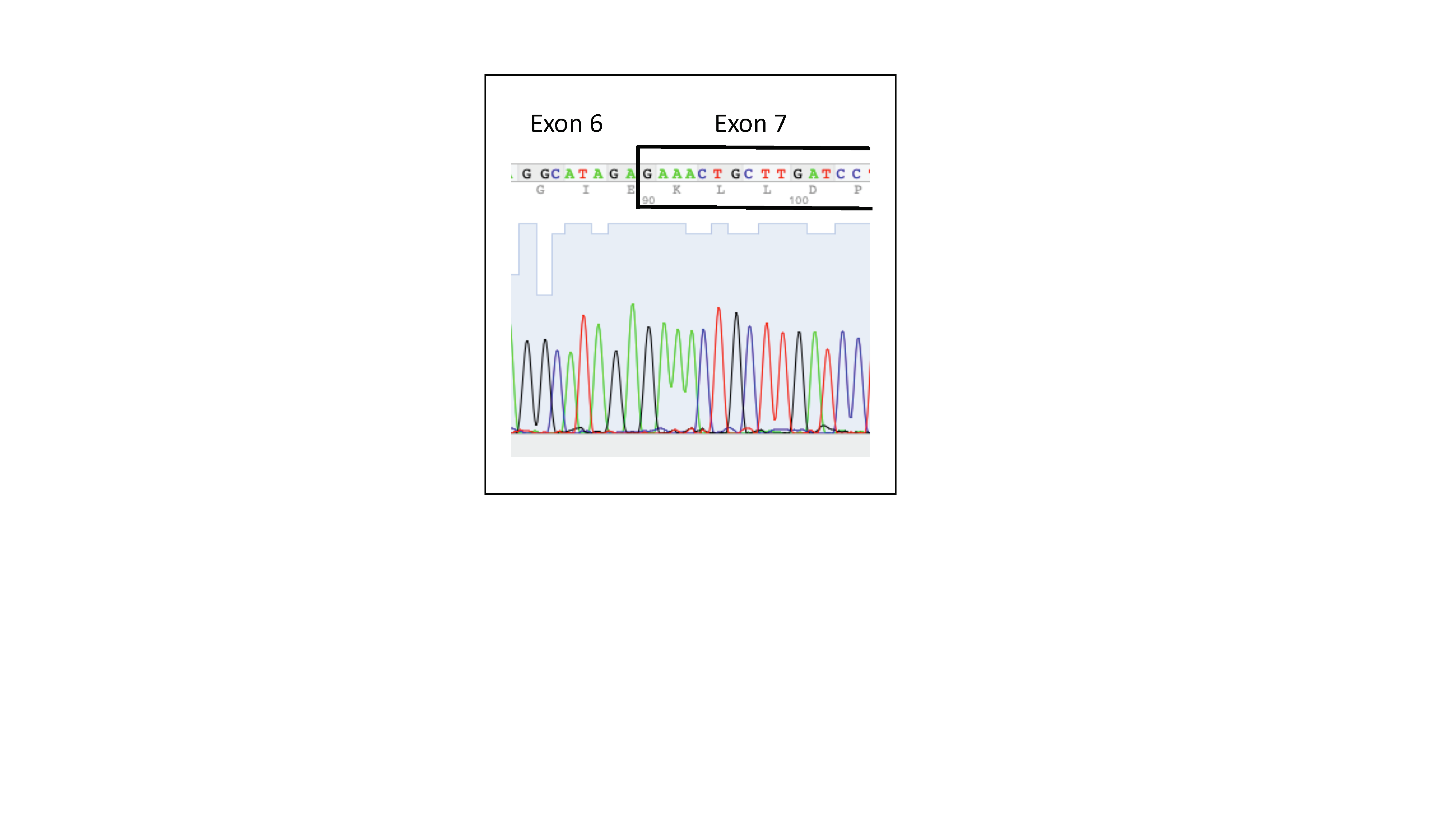

Supplement: S4 Fig — (a) Immunofluorescence microscopy: GRMD non-Tx cells had lower dystrophin expression compared to normal. Levels in sgRNA A-Tx, sgRNA B-Tx, sgRNA C, sgRNA C combined and donor clone only treated cells did not differ from normal, suggesting a potential treatment effect. However, this was not significantly different from non-Tx GRMD or normal cells. TALEN-Tx cells showed an increase in dystrophin protein when compared to non-Tx GRMD cells. Dystrophin expression for the two guides combined was significantly reduced compared to GRMD non-Tx and normal control. Intensity of dystrophin signal from multinucleated myotubes measured with ImageJ and analyzed via one way ANOVA. **** p ≤ 0.0001; * p ≤ 0.05. (b) Western blot: Dystrophin and β-spectrin signal for different treatments. β -spectrin was used as a loading control. (c) Western blot: Quantification of dystrophin signal normalized to normal myotubes protein extract. One way ANOVA was used and no statistical differences were found between treatments. Vertical bars indicate standard error of the mean. (TIFF) [file pone.0228072.s004.tiff]

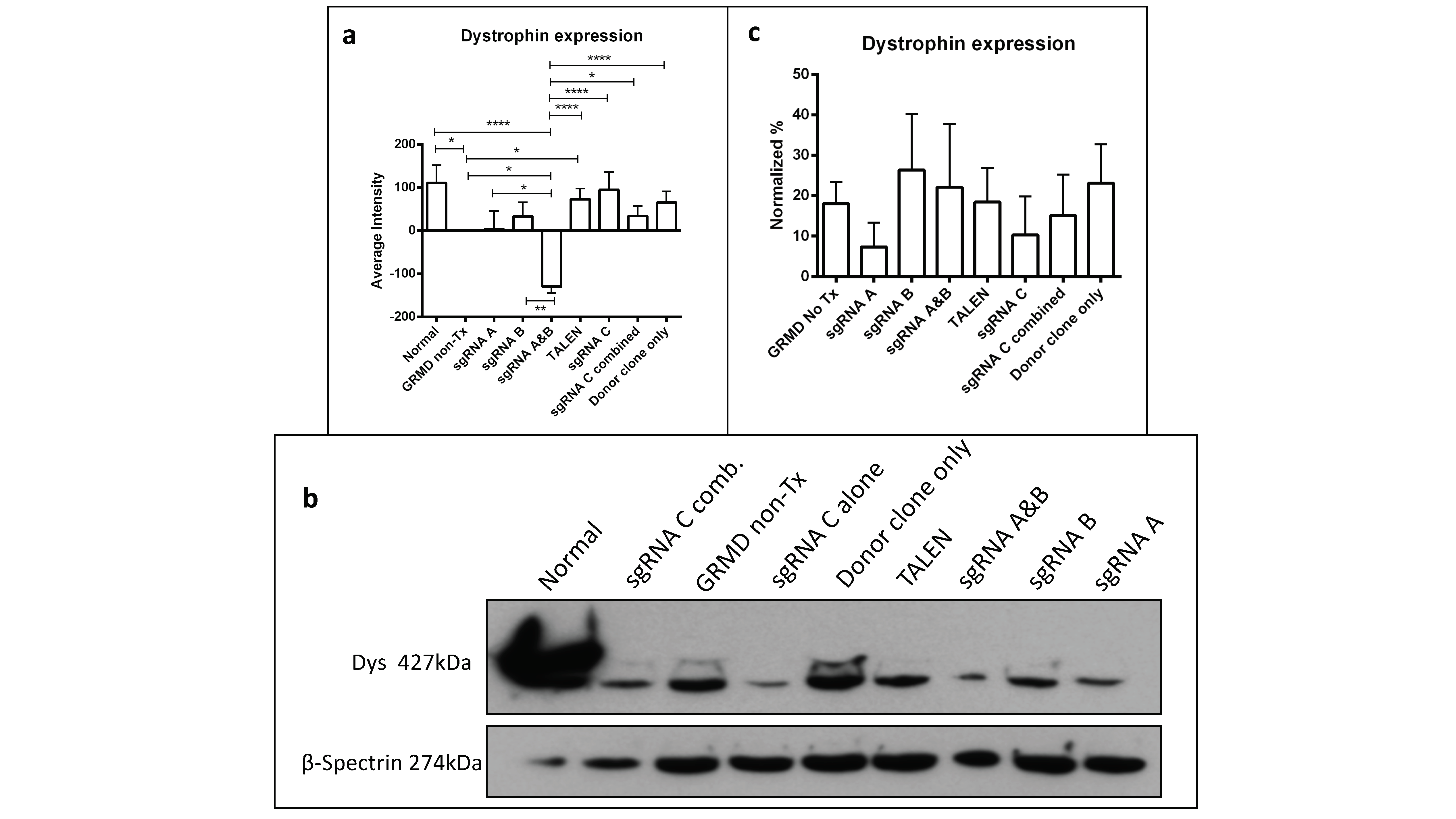

Supplement: S5 Fig — Exon 7 boundary area sequenced from HDR-CRISPR-Tx muscle. Exon 7 was included in the DMD mRNA of the gene edited muscle. (TIFF) [file pone.0228072.s005.tiff]

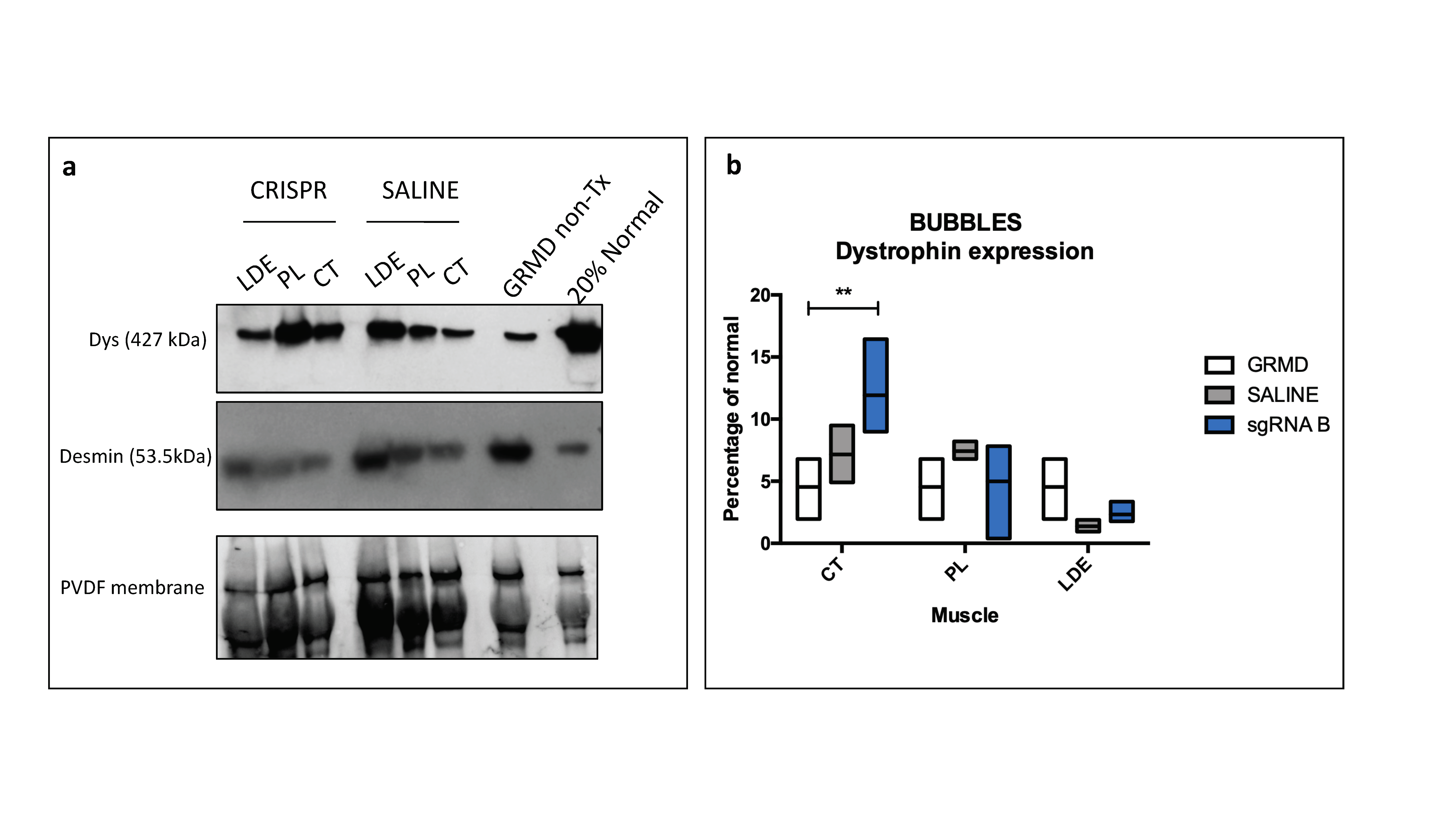

Supplement: S6 Fig — (a) Dystrophin co-stained with C and N-terminus antibodies with goat anti mouse secondary staining, desmin stained as a muscle marker with the same secondary staining. PVDF membrane as reference. (b) Graph with dystrophin quantification for each muscle in the cranial tibial compartment. PVDF membrane was used as a loading control and values were normalized by comparing them to 20% of dystrophin protein extract from normal dog CT muscle. Triplicate blots for each sample were used as replicates. Statistical analysis was performed with Tukey’s multiple comparison’s test ** p ≤ 0.01; CT = cranial tibial; Dys = dystrophin; LDE = long digital extensor; PL = peroneus longus; Tx = treatment. (TIFF) [file pone.0228072.s006.tiff]

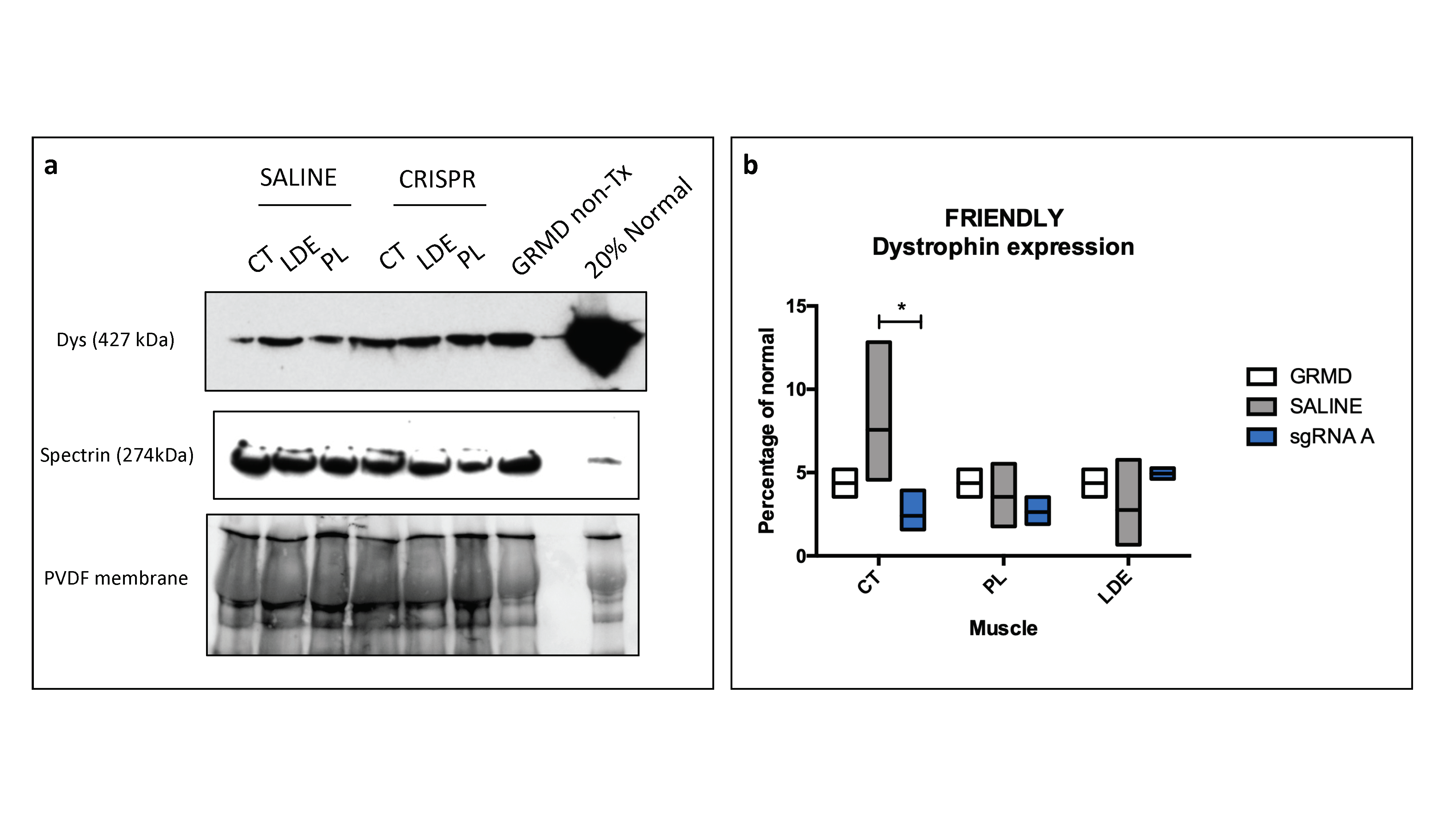

Supplement: S7 Fig — CRISPR sgRNA A was injected into one cranial tibial compartment and saline into the other. The biceps femoris (GRMD) was biopsied before injections and the HDR-CRISPR/saline muscles were harvested 3 months after treatment. (a) Dystrophin co-stained with C and N-terminus antibodies with goat anti mouse secondary staining, β-spectrin stained as a muscle marker. Total protein from the PVDF membrane was used to normalize dystrophin. Normal sample was diluted to 20%. (b) Graph with dystrophin quantification for each muscle in the cranial tibial compartment. Dystrophin in the saline-injected CT muscle was unexpectedly increased compared to the HDR-CRISPR-Tx CT muscle. Levels did not differ in the other HDR-CRISPR-Tx muscles. Statistical analysis was performed with Tukey’s multiple comparison’s test * p ≤ 0.05; CT = cranial tibial; LDE = long digital extensor; PL = peroneus longus. (TIFF) [file pone.0228072.s007.tiff]

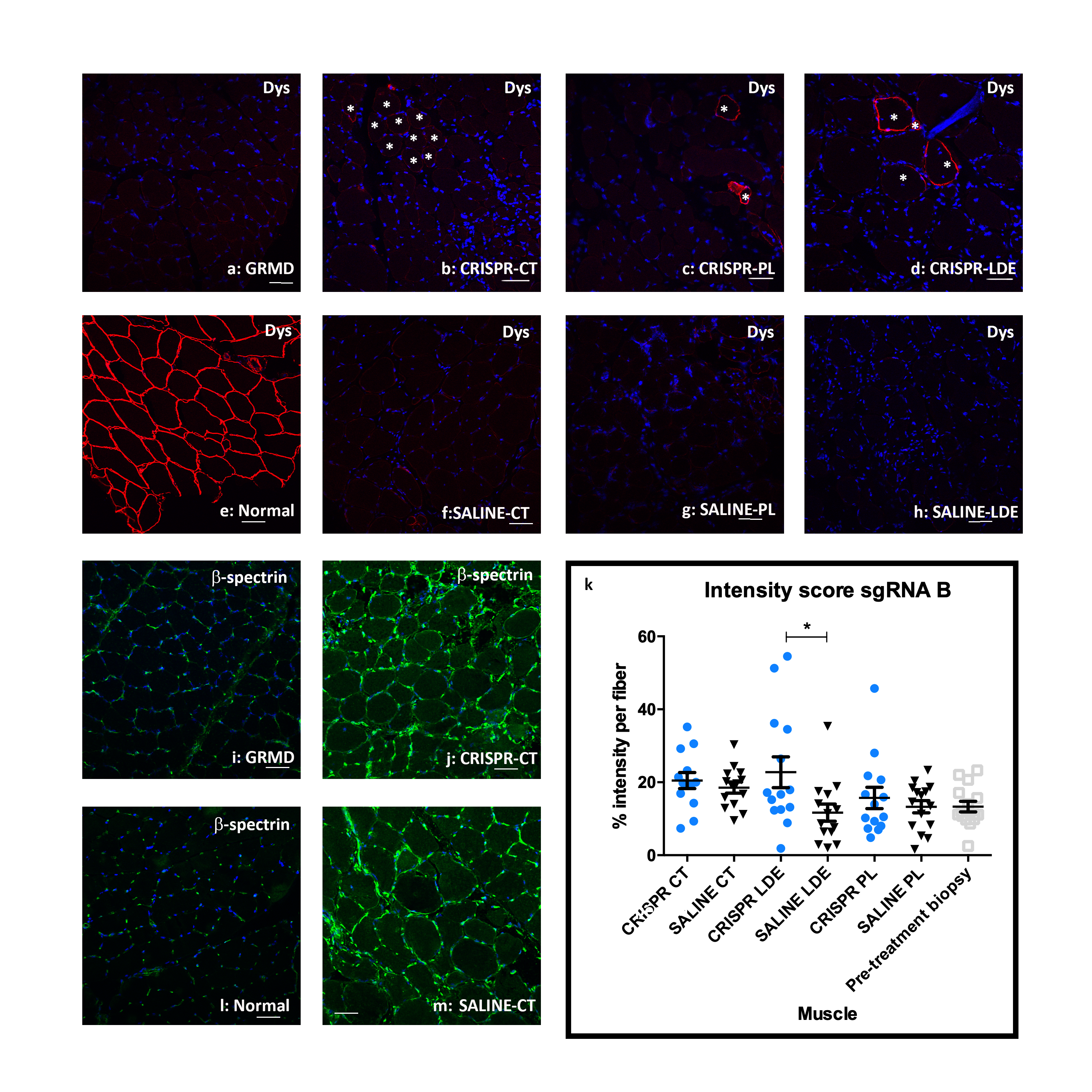

Supplement: S8 Fig — Dystrophin co-stained with C and N-terminus antibodies with Alexa 647 (red), β -spectrin membrane control (green) and DAPI denotes the nuclei (blue). Asterisks denotes cells with a value of 2 in intensity score for dystrophin signal in the GRMD non-Tx and Tx samples. Scale bar = 50μm. (a) Pre-treatment biopsy sample for Bubbles (b) HDR-CRISPR injected cranial tibial (CT) Bubbles (c) HDR-CRISPR injected peroneus longus (PL) Bubbles (d) HDR-CRISPR injected long digital extensor (LDE) Bubbles (e) normal dog muscle (f) SALINE injected CT Bubbles (g) SALINE injected PL Bubbles (h) SALINE injected LDE Bubbles. (i) Pre-treatment biopsy sample for Bubbles (j) HDR-CRISPR injected CT Bubbles (k) dystrophin intensity quantification for Bubbles and Friendly via One-way ANOVA multiple comparisons test, blue circle indicates CRISPR-Tx limb, black triangle indicates Saline-Tx limb, gray square indicates pre-treatment biopsied sample; *p<0.05 (l) normal dog muscle (m) SALINE injected CT Bubbles. Dys = dystrophin. (TIF) [file pone.0228072.s008.tif]

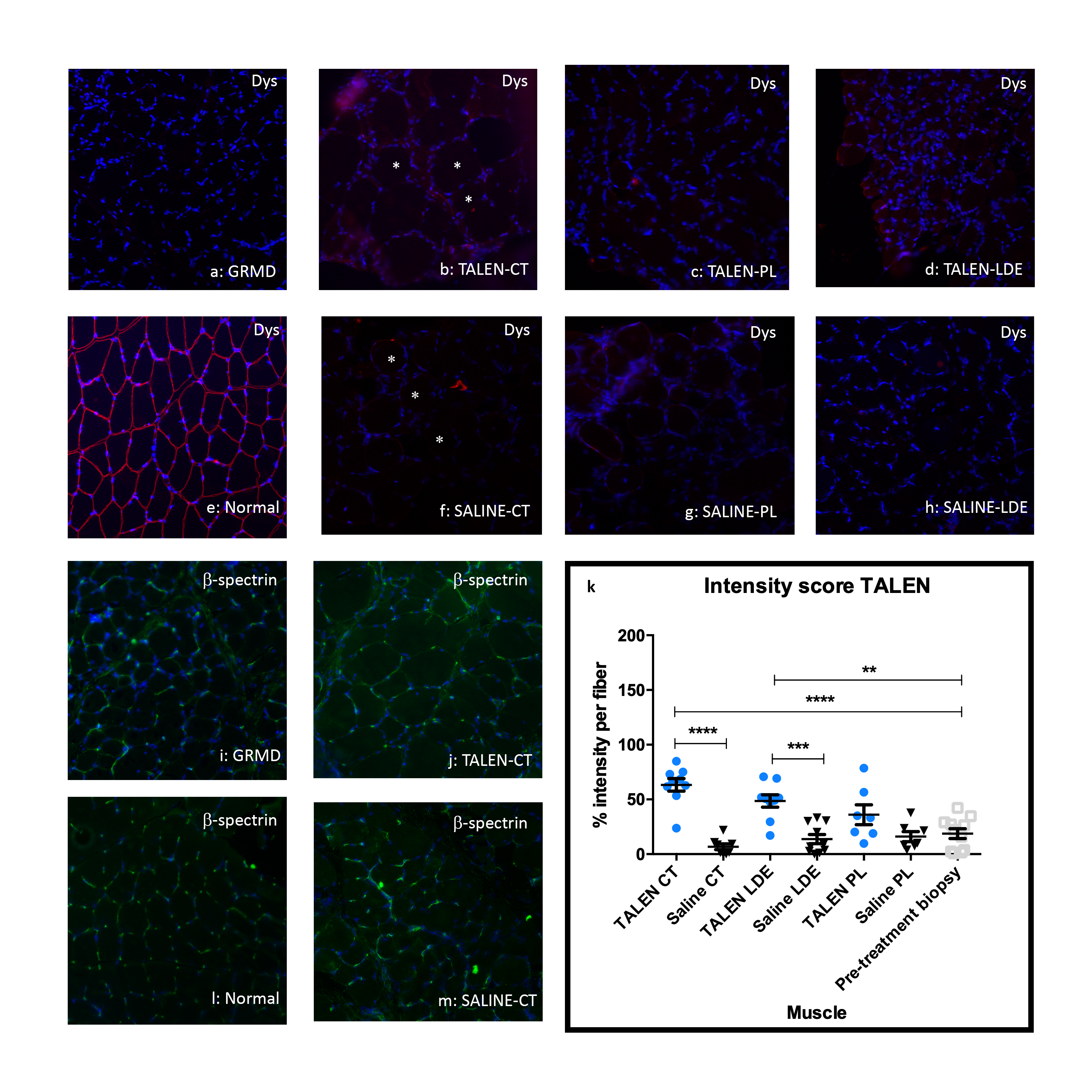

Supplement: S9 Fig — Dystrophin co-stained with C and N-terminus antibodies with Alexa 647 (red), β -spectrin membrane control (green) and DAPI denotes the nuclei (blue). Asterisks denotes cells with a value of 2 in intensity score for dystrophin signal in the GRMD non-Tx and Tx samples, dotted line denotes cells with a value of 1 in intensity score. Scale bar = 100μm. (a) Pre-treatment biopsy sample for Hera (b) HDR-CRISPR injected cranial tibial (CT) Hera (c) HDR-CRISPR injected peroneus longus (PL) Hera (d) HDR-CRISPR injected long digital extensor (LDE) Hera (e) normal dog muscle (f) SALINE injected CT Hera (g) SALINE injected PL Hera (h) SALINE injected LDE Hera. (i) Pre-treatment biopsy sample for Hera (j) HDR-CRISPR injected LDE Hera (k) dystrophin intensity quantification for Hera and Gantu via One-way ANOVA multiple comparisons test, blue circle indicates CRISPR-Tx limb, black triangle indicates Saline-Tx limb, gray square indicates pre-treatment biopsied sample; **p<0.01; ***p<0.001; ****p<0.0001 (l) normal dog muscle (m) SALINE injected LDE Hera. Dys = dystrophin. (TIF) [file pone.0228072.s009.tif]

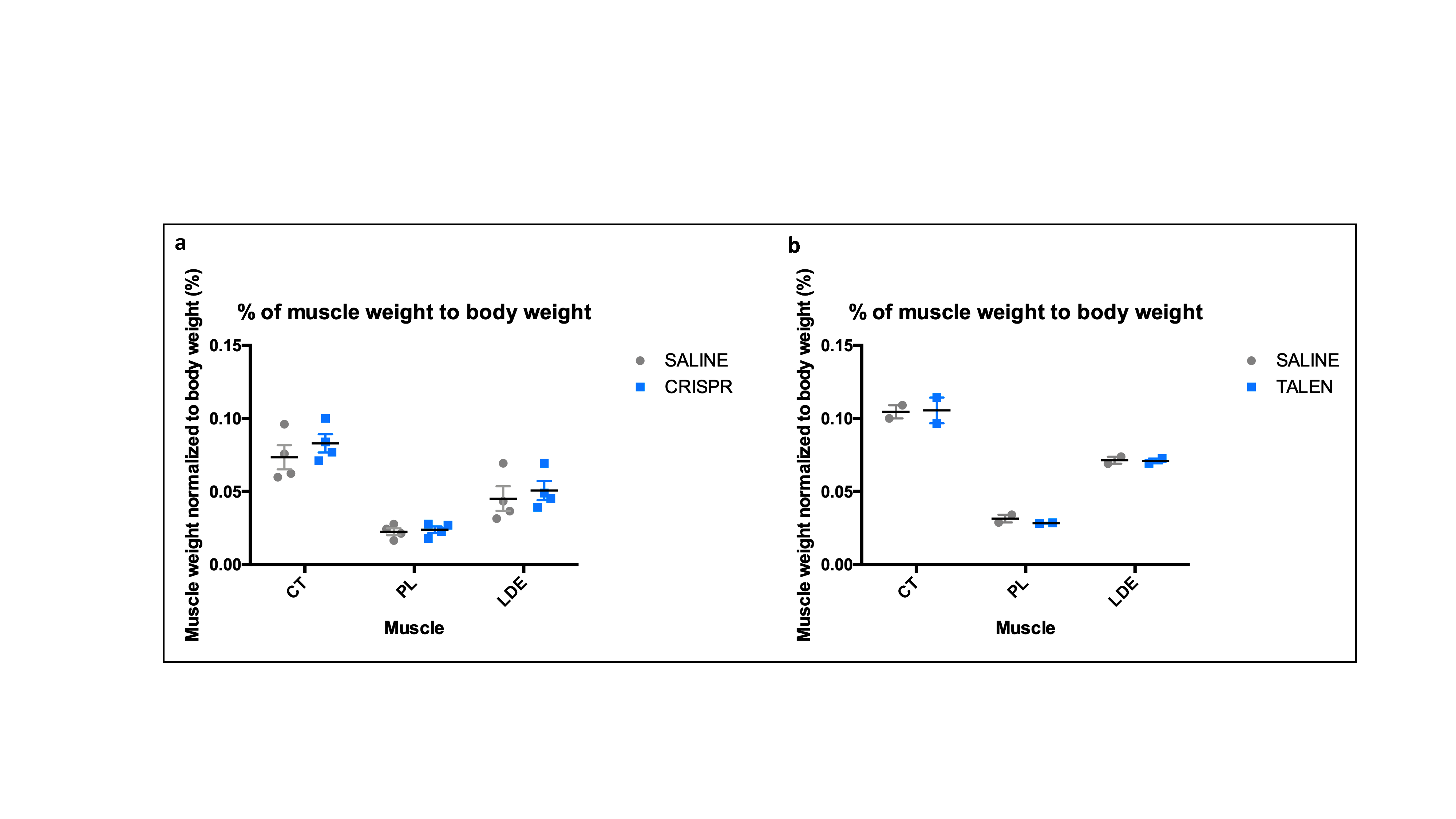

Supplement: S10 Fig — Cranial tibialis muscle (CT), peroneus longus (PL) and long digital extensor (LDE) were cleaned and weighed at necropsy. Muscle weights were normalized to the body weight of the dog and analyzed via One-way ANOVA. No significant differences were detected. Blue square indicates HDR-Tx and gray circle indicates Saline. (a) HDR-CRISPR treated dogs (b) HDR-TALEN treated dogs. (TIFF) [file pone.0228072.s010.tiff]

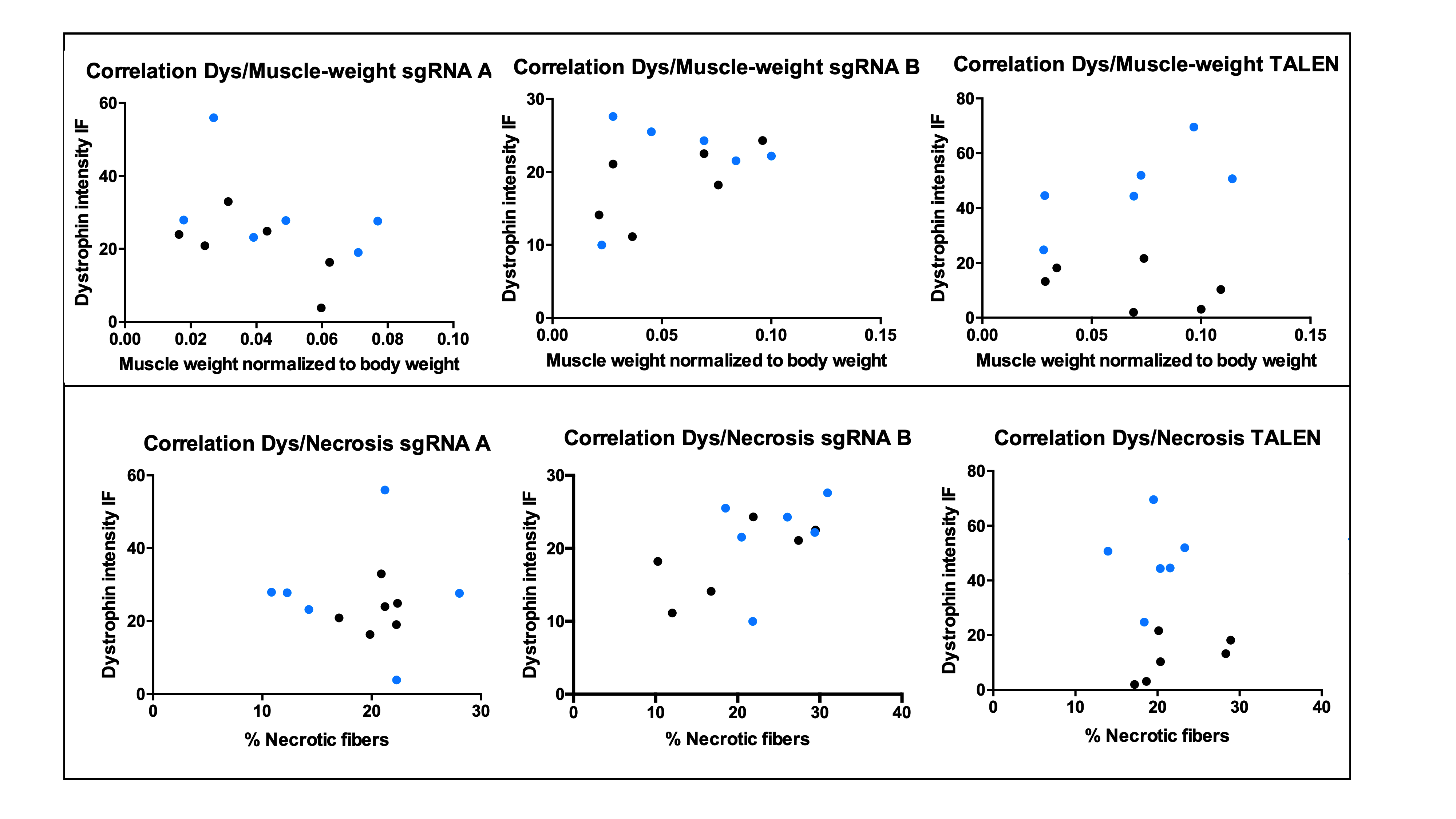

Supplement: S11 Fig — Correlations were studied between dystrophin IF single and muscle weight at necropsy (top row) and dystrophin IF signal and percentage of necrotic fibers (bottom row). Blue circle indicates HDR treated limb, black circle indicates saline treated limb. No correlations were found in any of the treatments studied. From left to right: sgRNA A, sgRNA B, TALEN. (TIFF) [file pone.0228072.s011.tiff]

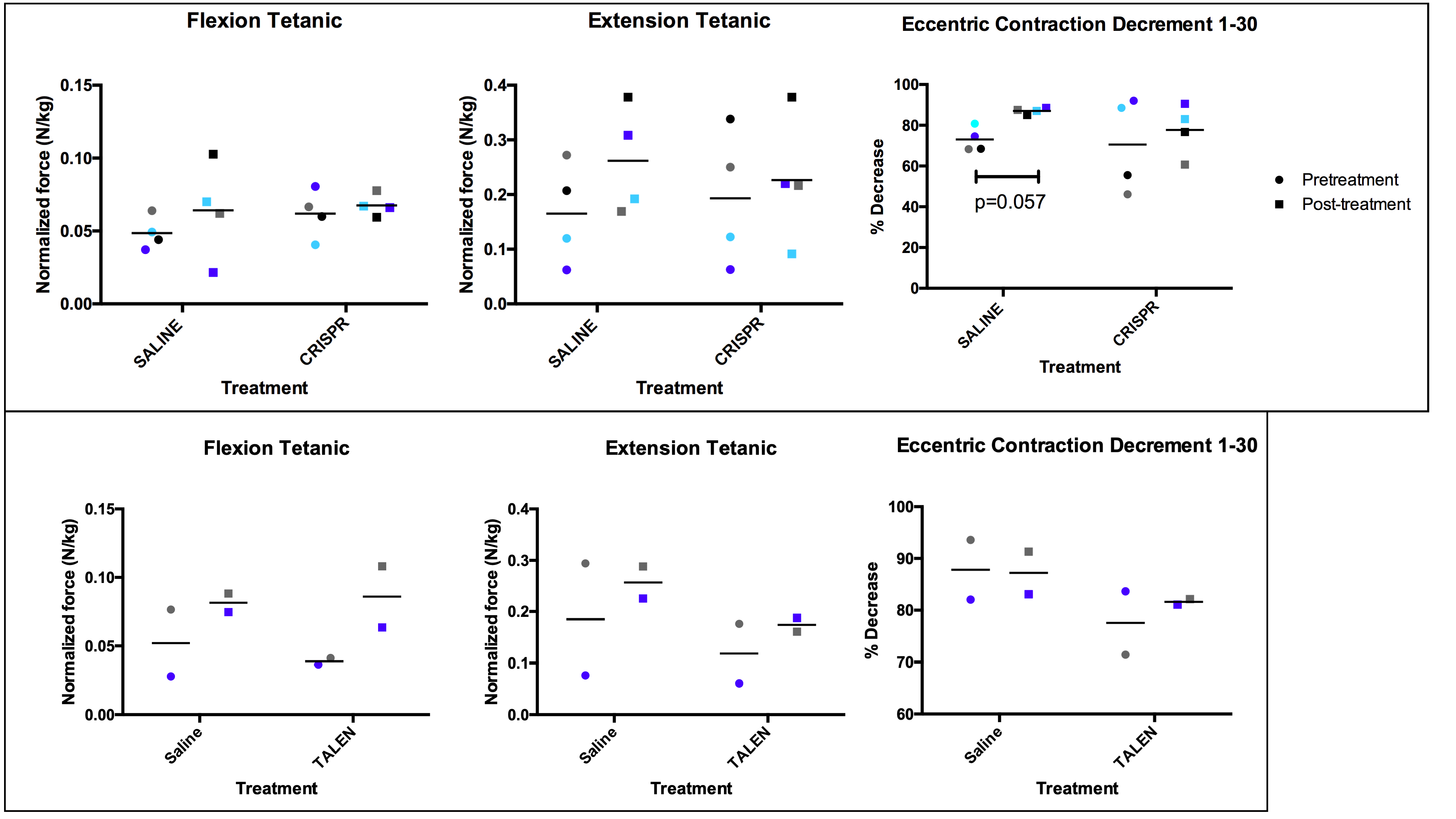

Supplement: S12 Fig — Circle is for pre-treatment values square is for post-treatment. Top: N = 4 analyzed via two way ANOVA. Blue color symbolizes sgRNA B’ data, grey color is for sgRNA A data. Bottom: N = 2 analyzed via two way ANOVA. From left to right: Extension tetanic values between saline and HDR- injected limbs as well as pretreatment and post-treatment. Flexion tetanic values. Eccentric contraction decrement (ECD). No statistical differences were found between saline and HDR-Tx limbs of GRMD dogs. In the saline injected limb for HDR-CRISPR ECD, there was a trend (p = 0.057) for an increase in ECD in post-treatment muscle compared to pre-treatment. The HDR-CRISPR injected limb ECD measurements were similar pre and post-treatment. (TIFF) [file pone.0228072.s012.tiff]
