# Supplementary material for: Challenges associated with homologous directed repair using CRISPR-Cas9 and TALEN to edit the DMD genetic mutation in canine Duchenne muscular dystrophy
Source: PLoS One. 2020 Jan 21;15(1):e0228072. doi: 10.1371/journal.pone.0228072 (PMC6974172; doi:10.1371/journal.pone.0228072)
Supplement: S1 Table — (DOCX) [file pone.0228072.s013.docx]

| Donor clone: |
| --- |
| 5’…gctcgaaattaaccctcactaaagggaacaaaagctggagctccaccgcggtggcggccttaattaagcgaattcgagatttgagaagtttatgatgaaacattttaacagatcactctgagagctttgcttagaatagattaacaagggcaaaggtgggagcagttggaaagttaatacaataaatactccatgcaagataaaacggtggtttagattgaggtgatggcagcaaggatagtgagaagtggtcaaattctggatatatttccaactaagtgttgtataagagtaagagtcagcaaattagactccaagatttctgtctgagccactgcaagtttaagcttgtccttgattattgaggaaaggagaagtaaaagaaaagtaggtgggttgggggagtgtgatggggatgaataccagaagcttggttttggacacctgaggcttatgatgactgtaagtggggatactgaataaacagatgaatctagagtctggagtttagggaagaagtctgggccaggcttataaaattggaacttaccagcaaatggctggcattcaaatttctgagactggatgacatcaatatattttttgttgcttagcaaggtagaataaattctcctttgttttcttcaggattttcaagtaaaattgtgacttgttaaatgaaaagtgttattgaggggtaataaagagtaacactcttaaggaatgatgggcagctagctgattgtcgagtaggacataacttcgtataatgtatgctatacgaagttatctgatcagatcctgcaggtactcgagGAACACACAAAAAACCAACACACAGATGTAATGAAAAATAAGATATTTTATTGCAACGCATGATACAAAGGCATTAAAGCAGCGTATCCACATAGCGTAAAAGGAGCAACATAGTTAAGAATACCAGTCAATCTTTCACAAATTTTGTAATCCAGAGGTTGATTAAGCTTTCAGAAGAACTCGTCAAGAAGGCGATAGAAGGCGATGCGCTGCGAATCGGGAGCGGCGATACCGTAAAGCACGAGGAAGCGGTCAGCCCATTCGCCGCCAAGCTCTTCAGCAATATCACGGGTAGCCAACGCTATGTCCTGATAGCGGTCCGCCACACCCAGCCGGCCACAGTCGATGAATCCAGAAAAGCGGCCATTTTCCACCATGATATTCGGCAAGCAGGCATCGCCATGGGTCACGACGAGATCCTCGCCGTCGGGCATGCGCGCCTTGAGCCTGGCGAACAGTTCGGCTGGCGCGAGCCCCTGATGCTCTTCGTCCAGATCATCCTGATCGACAAGACCGGCTTCCATCCGAGTACGTGCTCGCTCGATGCGATGTTTCGCTTGGTGGTCGAATGGGCAGGTAGCCGGATCAAGCGTATGCAGCCGCCGCATTGCATCAGCCATGATGGATACTTTCTCGGCAGGAGCAAGGTGAGATGACAGGAGATCCTGCCCCGGCACTTCGCCCAATAGCAGCCAGTCCCTTCCCGCTTCAGTGACAACGTCGAGCACAGCTGCGCAAGGAACGCCCGTCGTGGCCAGCCACGATAGCCGCGCTGCCTCGTCCTGCAGTTCATTCAGGGCACCGGACAGGTCGGTCTTGACAAAAAGAACCGGGCGCCCCTGCGCTGACAGCCGGAACACGGCGGCATCAGAGCAGCCGATTGTCTGTTGTGCCCAGTCATAGCCGAATAGCCTCTCCACCCAAGCGGCCGGAGAACCTGCGTGCAATCCATCTTGTTCAATCATAGGGCCGGGATTCTCCTCCACGTCACCGCATGTTAGAAGACTTCCTCTGCCCTCGCGAGATCCGGTGGAGCCGGGTCCGGCGGTGCCGTCCACGGCAGAATTGGACGACTGAGCGCGGGATCTGGCGAAGGCGATGGGGGTCTTGAAGGCGTGCTGGTACTCCACGATGCCCAGCTCGGTGTTGCTGTGCAGCTCCTCCACGCGGCGGAAGGCGAACATGGGGCCCCCGTTCTGCAGGATGCTGGGGTGGATGGCGCTCTTGAAGTGCATGTGGCTGTCCACCACGAAGCTGTAGTAGCCGCCGTCGCGCAGGCTGAAGGTGCGGGCGAAGCTGCCCACCAGCACGTTATCGCCCATGGGGTGCAGGTGCTCCACGGTGGCGTTGCTGCGGATGATCTTGTCGGTGAAGATCACGCTGTCCTCGGGGAAGCCGGTGCCCACCACCTTGAAGTCGCCGATCACGCGGCCGGCCTCGTAGCGGTAGCTGAAGCTCACGTGCAGCACGCCGCCGTCCTCGTACTTCTCGATGCGGGTGTTGGTGTAGCCGCCGTTGTTGATGGCGTGCAGGAAGGGGTTCTCGTAGCCGCTGGGGTAGGTGCCGAAGTGGTAGAAGCCGTAGCCCATCACGTGGCTCAGCAGGTAGGGGCTGAAGGTCAGGGCGCCTTTGGTGCTCTTCATCTTGTTGGTCATGCGGCCCTGCTTGGGGGTGCCCTCTCCGCCGCCCACCAGCTCGAACTCCACGCCGTTCAGGGTGCCGGTGATGCGGCACTCGATCTCCATGGCGGGCAGGCCGCTCTCGTCGCTCTCCATGGTGGCGTCTAGCGGTACCAGCTCTGCTTATATAGACCTCCCACCGTACACGCCTACCGCCCATTTGCGTCAATGGGGCGGAGTTGTTACGACATTTTGGAAAGTCCCGTTGATTTTGGTGCCAAAACAAACTCCCATTGACGTCAATGGGGTGGAGACTTGGAAATCCCCGTGAGTCAAACCGCTATCCACGCCCATTGATGTACTGCCAAAACCGCATCACCATGGTAATAGCGATGACTAATACGTAGATGTACTGCCAAGTAGGAAAGTCCCGTAAGGTCATGTACTGGGCATAATGCCAGGCGGGCCATTTACCGTCATTGACGTCAATAGGGGGCGGACTTGGCATATGATACACTTGATGTACTGCCAAGTGGGCAGTTTACCGTAAATACTCCACCCATTGACGTCAATGGAAAGTCCCTATTGGCGTTACTATGGGAACATACGTCATTATTGACGTCAATGGGCGGGGGTCGTTGGGCGGTCAGCCAGGCGGGCCATTTACCGTAAGTTATGTAACGCGGAACTCCATATATGGGCTATGAACTAATGACCCCGTAATTGATTACTATTAATAACTAGTCAATAATCAATGTCaacggatcctcatgatcgtagctacagctttgataacttcgtataatgtatgctatacgaagttataatctagaattgtaaattgatttatatttatttttatgtgtgtgtgtttcAgGCCAGACCTGTTTGATTGGAATAGTGTGGTTTGtCAGCAGTCAGCCACACAACGCCTGGAACATGCATTCAACATTGCCAAATATCAATTAGGCATAGAGAAACTGCTTGATCCTGAAGgtcggtacatttctggactaccatagtttttagtatagtttaatatttataatctcagacttaacatttctacaaactaagcatgaaagagaaactatgcattatatgtttttctatatccaggatgtatttaaaatgatgtcttcttgcagtgtattataaattgtcctatatggatttagttctcaaaatactattatctaaaactaaagtgtaaaacttgagaaaaaattttaagcctttagaattgtatacgcctcatgaatgaagcatgctacatttgaagtacattgactccaaatagttaaaaaaaagttcatgttttgcttattctaccaagagatttgatagactaatcagctataattaattagctactcttgccaatttcattcatatattctataaaaggcaacatggtttactgttgaaatcaggaaatctattcctggctctgcttctgttataatttgtcactctgggggaaatcctccattgaatggttattcattttctccttcttcagttgacaggcagcactgtatttatatttttattatattatgcttaagtaaatttcatcattatctctggtgctacatagtcatactttccagatacatggattagagggataatgattcttgacctctgcttttcttctagcctatgaggtactttttttgtttgccaacttgtttgaaagtcgacagcatttaaatcaattctaccgggtaggggaggcgcttttcccaaggcagtctggagcatgcgctttagcagccccgctggcacttggcgctacacaagtggcctctggcctcgcacacattccacatccaccggtagcgccaaccggctccgttctttggtggccccttcgcgccaccttctactcctcccctagtcaggaagttcccccccgccccgcagctcgcgtcgtgcaggacgtgacaaatggaagtagcacgtctcactagtctcgtgcagatggacagcaccgctgagcaatggaagcgggtaggcctttggggcagcggccaatagcagctttgctccttcgctttctgggctcagaggctgggaaggggtgggtccgggggcgggctcaggggcgggctcaggggcggggcgggcgcgaaggtcctcccgaggcccggcattctcgcacgcttcaaaagcgcacgtctgccgcgctgttctcctcttcctcatctccgggcctttcgacctgcagcgacccgcttaacagcgtcaacagcgtgccgcagatcttggtggcgtgaaactcccgcacctcttcggccagcgccttgtagaagcgcgtatggcttcgtacccctgccatcaacacgcgtctgcgttcgaccaggctgcgcgttctcgcggccataacaaccgacgtacggcgttgcgccctcgccggcaacaaaaagccacggaagtccgcctggagcagaaaatgcccacgctactgcgggtttatatagacggtccccacgggatggggaaaaccaccaccacgcaactgctggtggccctgggttcgcgcgacgatatcgtctacgtacccgagccgatgacttactggcgggtgttgggggcttccgagacaatcgcgaacatctacaccacacaacaccgcctcgaccagggtgagatatcggccggggacgcggcggtggtaatgacaagcgcccagataacaatgggcatgccttatgccgtgaccgacgccgttctggctcctcatatcgggggggaggctgggagctcacatgccccgcccccggccctcaccctcatcttcgaccgccatcccatcgccgccctcctgtgctacccggccgcgcgataccttatgggcagcatgaccccccaggccgtgctggcgttcgtggccctcatcccgccgaccttgcccggcacaaacatcgtgttgggggcccttccggaggacagacacatcgaccgcctggccaaacgccagcgccccggcgagcggcttgacctggctatgctggccgcgattcgccgcgtttatgggctgcttgccaatacggtgcggtatctgcagggcggcgggtcgtggcgggaggattggggacagctttcgggggcggccgtgccgccccagggtgccgagccccagagcaacgcgggcccacgaccccatatcggggacacgttatttaccctgtttcgggcccccgagttgctggcccccaacggcgacctgtacaacgtgtttgcctgggccttggacgtcttggccaaacgcctccgtcccatgcacgtctttatcctggattacgaccaatcgcccgccggctgccgggacgccctgctgcaacttacctccgggatggtccagacccacgtcaccacccccggctccataccgacgatctgcgacctggcgcgcacgtttgcccgggagatgggggaggctaactgaaacacggaaggagacaataccggaaggaacccgcgctatgacggcaataaaaagacagaataaaacgcacgggtgttgggtcgtttgttcataaacgcggggttcggtcccagggctggcactctgtcgataccccaccgagaccccattggggccaatacgcccgcgtttcttccttttccccaccccaccccccaagttcgggtgaaggcccagggctcgcagccaacgtcggggcggcaagccctgccatagccacgggccccgtgggttagggacggggtcccccatggggaatggtttatggttcgtgggggttattattttgggcgttgcgtggggtcaggtccacgactggactgagcagacagacccatggtttttggatggcctgggcatggaccgcatgtactggcgcgacacgaacaccgggcgtctgtggctgccaaacacccccgacccccaaaaaccaccgcgcggatttctggcgccgccggacgaactaaacctgactacggcatctctgccccttcttcgctggtacgaggagcgcttttgttttgtattggtcaccacggccgagtttccgcgggaccccggccaggacctgcagaaattgatgatctattaaacaataaagatgtccactaaaatggaagtttttcctgtcatactttgttaagaagggtgagaacagagtacctacattttgaatggaaggattggagctacgggggtgggggtggggtgggattagataaatgcctgctctttactgaaggctctttactattgctttatgataatgtttcatagttggatatcataatttaaacaagcaaaaccaaattaagggccagctcattcctcccactcatgatctatagatctatagatctctcgtgggatcattgtttttctcttgattcccactttgtggttctaagtactgtggtttccaaatgtgtcagtttcatagcctgaagaacgagatcagcagcctctgttccacatacacttcattctcagtattgttttgccaagttctaattccatcagaagcttggcactggccgtcgttttacaacgtcgtgactgggaaaaccctggcgttacccaacttaatcgccttgcagcacatccccctttcgccagctggcgtaatagcgaagaggcccgcaccgatcgcccttcccaacagttgcgcagcctgaatggcgaatggcgcctgatgcggtattttctccttacgcatctgtgcggtatttcacaccgcatatggtgcactctcagtacaatctgctctgatgccgcatagttaagccagccccgacacccgccaacacccgctgacgcgccctgacgggcttgtctgctcccggcatccgcttacagacaagctgtgaccgtctccgggagctgcatgtgtcagaggttttcaccgtcatcaccgaaacgcgcgagacgaaagggcctcgtgatacgcctatttttataggttaatgtcatgataataatggtttcttagacgtcaggtggcacttttcggggaaatgtgcgcggaacccctatttgtttatttttctaaatacattcaaatatgtatccgctcatgagacaataaccctgataaatgcttcaataatattgaaaaaggaagagtatgagtattcaacatttccgtgtcgcccttattcccttttttgcggcattttgccttcctgtttttgctcacccagaaacgctggtgaaagtaaaagatgctgaagatcagttgggtgcacgagtgggttacatcgaactggatctcaacagcggtaagatccttgagagttttcgccccgaagaacgttttccaatgatgagcacttttaaagttctgctatgtggcgcggtattatcccgtattgacgccgggcaagagcaactcggtcgccgcatacactattctcagaatgacttggttgagtactcaccagtcacagaaaagcatcttacggatggcatgacagtaagagaattatgcagtgctgccataaccatgagtgataacactgcggccaacttacttctgacaacgatcggaggaccgaaggagctaaccgcttttttgcacaacatgggggatcatgtaactcgccttgatcgttgggaaccggagctgaatgaagccataccaaacgacgagcgtgacaccacgatgcctgtagcaatggcaacaacgttgcgcaaactattaactggcgaactacttactctagcttcccggcaacaattaatagactggatggaggcggataaagttgcaggaccacttctgcgctcggcccttccggctggctggtttattgctgataaatctggagccggtgagcgtgggtctcgcggtatcattgcagcactggggccagatggtaagccctcccgtatcgtagttatctacacgacggggagtcaggcaactatggatgaacgaaatagacagatcgctgagataggtgcctcactgattaagcattggtaactgtcagaccaagtttactcatatatactttagattgatttaaaacttcatttttaatttaaaaggatctaggtgaagatcctttttgataatctcatgaccaaaatcccttaacgtgagttttcgttccactgagcgtcagaccccgtagaaaagatcaaaggatcttcttgagatcctttttttctgcgcgtaatctgctgcttgcaaacaaaaaaaccaccgctaccagcggtggtttgtttgccggatcaagagctaccaactctttttccgaaggtaactggcttcagcagagcgcagataccaaatactgtccttctagtgtagccgtagttaggccaccacttcaagaactctgtagcaccgcctacatacctcgctctgctaatcctgttaccagtggctgctgccagtggcgataagtcgtgtcttaccgggttggactcaagacgatagttaccggataaggcgcagcggtcgggctgaacggggggttcgtgcacacagcccagcttggagcgaacgacctacaccgaactgagatacctacagcgtgagctatgagaaagcgccacgcttcccgaagggagaaaggcggacaggtatccggtaagcggcagggtcggaacaggagagcgcacgagggagcttccagggggaaacgcctggtatctttatagtcctgtcgggtttcgccacctctgacttgagcgtcgatttttgtgatgctcgtcaggggggcggagcctatggaaaaacgccagcaacgcggcctttttacggttcctggccttttgctggccttttgctcacatgttctttcctgcgttatcccctgattctgtggataaccgtattaccgcctttgagtgagctgataccgctcgccgcagccgaacgaccgagcgcagcgagtcagtgagcgaggaagcggaagagcgcccaatacgcaaaccgcctctccccgcgcgttggccgattcattaatgcagctggcacgacaggtttcccgactggaaagcgggcagtgagcgcaacgcaattaatgtgagttagctcactcattaggcaccccaggctttacactttatgcttccggctcgtatgttgtgtggaattgtgagcggataacaatttcacacaggaaacagctatgaccatgattacgccaa…3’ |
