# Supplementary material for: Challenges associated with homologous directed repair using CRISPR-Cas9 and TALEN to edit the DMD genetic mutation in canine Duchenne muscular dystrophy
Source: PLoS One. 2020 Jan 21;15(1):e0228072. doi: 10.1371/journal.pone.0228072 (PMC6974172; doi:10.1371/journal.pone.0228072)
Supplement: S2 Table — (DOCX) [file pone.0228072.s014.docx]

| Donor clone forward | 5’…TTCACACAGGAAACAGCTATGACC…3’ |
| --- | --- |
| Donor clone reverse | 5’…CCTGGCCCAGACTTCTTCCCTAA…3’ |
| Left TALEN forward | 5’…TAGTGGCTATTGCATCCAACATC…3’ |
| Left TALEN reverse | 5’…AACTCGGTCACTGATGAAGGATA…3’ |
| Right TALEN forward | 5’…CCAGTTGCTGAAGATCGCGAAGC …3’ |
| Right TALEN reverse | 5’…CGACTTGTTCGGGTGTAAGT …3’ |
| sgRNA A forward | 5’…CGCTCTTAAGGAATGATGGGCA…3’ |
| sgRNA B forward | 5’…GTTGTGTGGCTGACTGCGT…3’ |
| sgRNA A and B reverse | 5’…AGTCCCTATTGGCGTTACTATG…3’ |
