# Supplementary material for: Challenges associated with homologous directed repair using CRISPR-Cas9 and TALEN to edit the DMD genetic mutation in canine Duchenne muscular dystrophy
Source: PLoS One. 2020 Jan 21;15(1):e0228072. doi: 10.1371/journal.pone.0228072 (PMC6974172; doi:10.1371/journal.pone.0228072)
Supplement: S3 Table — (DOCX) [file pone.0228072.s015.docx]

| Gene specific primer | 5’…AGCTGAAAGAAGCCACGAGA…3’ |
| --- | --- |
| Outside forward | 5’…GTCTGATGGCCTGGCTTTGA…3’ |
| Outside reverse | 5’…TCCAGGTTTGCTTCGGTCTC…3’ |
| Inside forward | 5’…CCACAGTCATAGGCCAGACC…’3 |
| Inside reverse | 5’…GCCCTGTGCTAGACTGACTG…3’ |
