# Supplementary material for: Challenges associated with homologous directed repair using CRISPR-Cas9 and TALEN to edit the DMD genetic mutation in canine Duchenne muscular dystrophy
Source: PLoS One. 2020 Jan 21;15(1):e0228072. doi: 10.1371/journal.pone.0228072 (PMC6974172; doi:10.1371/journal.pone.0228072)
Supplement: S4 Table — (DOCX) [file pone.0228072.s016.docx]

| Dystrophin forward (exon 28) | 5’…CTGGGGGAGCTGAGGAAATC…3’ |
| --- | --- |
| Dystrophin reverse (exon 29) | 5’…CGGGTTATCCTCTGAATGTTGC…3’ |
| HPRT1 forward | 5’…AGCTTGCTGGTGAAAAGGAC…3’ |
| HPRT1 reverse | 5’…TTATAGTCAAGGGCATATCC…3’ |
