# Supplementary material for: Challenges associated with homologous directed repair using CRISPR-Cas9 and TALEN to edit the DMD genetic mutation in canine Duchenne muscular dystrophy
Source: PLoS One. 2020 Jan 21;15(1):e0228072. doi: 10.1371/journal.pone.0228072 (PMC6974172; doi:10.1371/journal.pone.0228072)
Supplement: S5 Table — Capitalized letters indicate matching sequences to the predicted off target DNA location. (DOCX) [file pone.0228072.s017.docx]

| Treatment | Target | Chr. | Position | Strain | Mismatches |
| --- | --- | --- | --- | --- | --- |
| sgRNA A&B, sgRNA A | DNA:  tTCTgAtGGAATGATGGGCATGG | 24 | 33445254 | - | 3 |
| sgRNA A&B, sgRNA A | DNA:  CTCTTAAaGAATGATGGtCtGGG | 7 | 55297606 | - | 3 |
| sgRNA A&B, sgRNA A | DNA:  CTCTTcAaGAATGATGGtCAAGG | 7 | 70903807 | + | 3 |
| sgRNA A&B, sgRNA B | DNA:  GGCcTgGTtTGGCTGATGCAGG | 7 | 45262680 | + | 3 |
| sgRNA A&B, sgRNA A | DNA: tgCTgAAGGAATGATGGGCAGGG | 4 | 25850139 | + | 3 |
| sgRNA A&B, sgRNA B | DNA:  GGCcTTGTGTGtCTGgCTGTCGG | 4 | 60719364 | + | 3 |
| sgRNA A&B, sgRNA A | DNA:  CTCTTAAGctATGATGGaCATGG | 31 | 13301286 | + | 3 |
| sgRNA A&B, sgRNA A | DNA:  CTCTgAAGGAgTGAgGGGCATGG | 31 | 39084604 | - | 3 |
| sgRNA A&B, sgRNA A | DNA:  CTCTTcAGGAATGAaGtGCAAGG | 5 | 20189533 | - | 3 |
| sgRNA B | DNA:  GGCGTTGacTGGCTGACTGaGGG | 37 | 23304219 | - | 3 |
| sgRNA B | DNA:  GGaGTTGTGTGGgTtACTGCAGG | 33 | 5028749 | + | 3 |
| sgRNA B | DNA:  GagGaTGTGTGGCTGACTGCAGG | 10 | 22022810 | + | 3 |
| sgRNA B | DNA:  GGCGaTGcGTGGCTGgCTGCCGG | 9 | 51699172 | - | 3 |
| sgRNA B | DNA:  GcCtTTGTGTGGCTGACTtCAGG | 11 | 39446025 | - | 3 |
| sgRNA B | DNA:  GGCaTgGTGTGtCTGACTGCAGG | 11 | 54163377 | + | 3 |
