# Supplementary material for: Challenges associated with homologous directed repair using CRISPR-Cas9 and TALEN to edit the DMD genetic mutation in canine Duchenne muscular dystrophy
Source: PLoS One. 2020 Jan 21;15(1):e0228072. doi: 10.1371/journal.pone.0228072 (PMC6974172; doi:10.1371/journal.pone.0228072)
Supplement: S6 Table — (DOCX) [file pone.0228072.s018.docx]

| Forward primer 5’…3’ | Reverse primer 5’…3’ | Chr. | Position |
| --- | --- | --- | --- |
| GCCAATGGCACCAAACTGC | ATAGCGAGGAAACCAGCGTG | 24 | 33445254 |
| TGATGCTTTCCTACCTCACT | TGGGGAAAACATGACAGATGC | 7 | 55297606 |
| TGATTTCACCCCACTAGCCC | AGCTTAAGGATGAAGGGTGAT | 7 | 70903807 |
| CTGCTGGCTTTAAGTAGCGT | AGGAGGCAAGACTGATGGGA | 7 | 45262680 |
| TCCAGTGGAAGGGCAGTTTG | GATGGCTTAAGGCTTCTGCTG | 4 | 25850139 |
| CAGGAAGCAGATCCATGGGG | TGCTCATTTTGTGGGTCCCT | 4 | 60719364 |
| GCTGTAAGCGCGTGTTCAAT | ATCAGCTCCTACAGCCTCCT | 31 | 13301286 |
| GAGCCACTTGCTCACACTCT | GAAATCAGGGAAGGCCCCAA | 31 | 39084604 |
| AGTAAAACTGGCAGGGGACT | GTAGGGAAGCGGCCTCA | 5 | 20189533 |
| TCCCCTCACCCCCAAGAAAG | TTGCGATGTTTGAGTGCTGG | 37 | 23304219 |
| TAGTCTGGGAGCATCAGCCA | ATCAGTGACCCCCAGTGTTG | 33 | 5028749 |
| GGGAAACATGCAGGGAGTGA | AGGAAGAGAGGCCCTTGGAA | 10 | 22022810 |
| GAGCCACAGTACACACCACC | ACATCCATCAGCCGTGACAG | 9 | 51699172 |
| GCACACCTTGGGAAAGTCAAC | CTCTAGCATCACCTCCGCTT | 11 | 39446025 |
| GGAGACTGCCAAGGTAAACAAAT | CTGTACTCCTAGAGGGGAGGT | 11 | 54163377 |
