# Supplementary material for: Challenges associated with homologous directed repair using CRISPR-Cas9 and TALEN to edit the DMD genetic mutation in canine Duchenne muscular dystrophy
Source: PLoS One. 2020 Jan 21;15(1):e0228072. doi: 10.1371/journal.pone.0228072 (PMC6974172; doi:10.1371/journal.pone.0228072)
Supplement: S7 Table — Treated myoblasts were differentiated into myotubes for 18 to 21 days and RNA was extracted from 6 replicates, values were normalized to HPRT1 (house-keeping gene). Exons 28–29 were targeted for QRT-PCR. Fold change was calculated compared to the column treatment with the cells with gray backslash. F.change = fold change; SE = standard error; ***p≤ 0.001; ** p ≤ 0.01; * p ≤ 0.05. Samples were analyzed using a pair wise fixed reallocation randomization test, excluding outliers with a Grubb’s test. Sg RNA C combined denotes when SgRNA C was combined with donor clone. (DOCX) [file pone.0228072.s019.docx]

|  | **Normal** | **sgRNA A** | **sgRNA B** | **sgRNA A&B** | **TALEN** | **sgRNA C** | **sgRNA C combined** | **Donor clone** | **GRMD No-Tx** |
| --- | --- | --- | --- | --- | --- | --- | --- | --- | --- |
| **F.change** |  | 2.739 | 2.496 | -4.283 | -2.211 | 8.794 | -1.336 | 6.328 | 6.434 |
| **SE** |  | ±2.519 | ±1.793 | ±0.337 | ±0.353 | ±12.792 | ±0.600 | ±7.395 | ±4.997 |
| **p-value** |  | ** | ** | *** | *** | ** | 0.12 | ** | ** |
|  | **Normal** | **sgRNA A** | **sgRNA B** | **sgRNA A&B** | **TALEN** | **sgRNA C** | **sgRNA C combined** | **Donor clone** | **GRMD No-Tx** |
| **F.change** | -2.739 |  | -1.097 | -11.732 | -6.057 | 3.21 | -3.659 | 2.31 | 2.349 |
| **SE** | ±0.320 |  | ±0.4997 | ±0.103 | ±0.105 | ±4.3193 | ±0.173 | ±2.436 | ±1.401 |
| **p-value** | ** |  | 0.625 | *** | *** | 0.069 | *** | 0.053 | * |
|  | **Normal** | **sgRNA A** | **sgRNA B** | **sgRNA A&B** | **TALEN** | **sgRNA C** | **sgRNA C combined** | **Donor clone** | **GRMD No-Tx** |
| **F.change** | -2.496 | 1.097 |  | -10.692 | -5.52 | 3.523 | -3.335 | 2.535 | 2.577 |
| **SE** | ±0.288 | ±0.631 |  | ±0.083 | ±0.075 | ±4.431 | ±0.096 | ±2.377 | ±0.531 |
| **p-value** | ** | 0.625 |  | * | *** | * | * | * | *** |
|  | **Normal** | **sgRNA A** | **sgRNA B** | **sgRNA A&B** | **TALEN** | **sgRNA C** | **sgRNA C combined** | **Donor clone** | **GRMD No-Tx** |
| **F.change** | 4.283 | 11.732 | 10.692 |  | 1.937 | 37.665 | 3.206 | 27.102 | 27.554 |
| **SE** | ±2.373 | ±5.750 | ±3.669 |  | ±0.781 | ±32.053 | ±1.275 | ±18.065 | ±10.301 |
| **p-value** | *** | *** | * |  | * | *** | *** | * | *** |
|  | **Normal** | **sgRNA A** | **sgRNA B** | **sgRNA A&B** | **TALEN** | **sgRNA C** | **sgRNA C combined** | **Donor clone** | **GRMD No-Tx** |
| **F.change** | 2.211 | 6.057 | 5.52 | -1.937 |  | 19.446 | 1.655 | 13.993 | 14.226 |
| **SE** | ±1.934 | ±4.550 | ±2.5541 | ±0.606 |  | ±27.278 | ±0.935 | ±15.106 | ±7.252 |
| **p-value** | ******* | *** | *** | * |  | *** | *** | ** | *** |
|  | **Normal** | **sgRNA A** | **sgRNA B** | **sgRNA A&B** | **TALEN** | **sgRNA C** | **sgRNA C combined** | **Donor clone** | **GRMD No-Tx** |
| **F.change** | -8.794 | -3.21 | -3.523 | -37.665 | -1.655 |  | -11.748 | -1.39 | -1.367 |
| **SE** | ±0.151 | ±0.402 | ±0.326 | ±0.053 | ±0.059 |  | ±0.105 | ±1.042 | ±0.903 |
| **p-value** | ** | 0.069 | * | *** | *** |  | *** | 0.535 | 0.502 |
|  | **Normal** | **sgRNA A** | **sgRNA B** | **sgRNA A&B** | **TALEN** | **sgRNA C** | **sgRNA C combined** | **Donor clone** | **GRMD No-Tx** |
| **F.change** | 1.336 | 3.659 | 3.335 | -3.206 | -1.655 | 11.748 |  | 8.453 | 8.594 |
| **SE** | ±0.975 | ±2.213 | ±0.971 | ±0.294 | ±0.278 | ±14.417 |  | ±7.843 | ±2.843 |
| **p-value** | 0.12 | *** | * | *** | *** | *** |  | ** | *** |
|  | **Normal** | **sgRNA A** | **sgRNA B** | **sgRNA A&B** | **TALEN** | **sgRNA C** | **sgRNA C combined** | **Donor clone** | **GRMD No-Tx** |
| **F.change** | -6.328 | -2.31 | -2.535 | -27.102 | -13.993 | 1.39 | -8.453 |  | 1.017 |
| **SE** | ±0.186 | ±0.481 | ±0.371 | ±0.064 | ±0.069 | ±2.213 | ±0.121 |  | ±1.031 |
| **p-value** | ** | 0.053 | * | * | ** | 0.535 | ** |  | 0.963 |
|  | **Normal** | **sgRNA A** | **sgRNA B** | **sgRNA A&B** | **TALEN** | **sgRNA C** | **sgRNA C combined** | **Donor clone** | **GRMD No-Tx** |
| **F.change** | -6.434 | -2.349 | -2.577 | -27.554 | -14.226 | 1.367 | -8.594 | -1.017 |  |
| **SE** | ±0.105 | ±0.233 | ±0.070 | ±0.031 | ±0.028 | ±1.615 | ±0.037 | ±0.868 |  |
| **p-value** | ** | * | *** | *** | *** | 0.502 | *** | 0.963 |  |
