# Supplementary material for: Challenges associated with homologous directed repair using CRISPR-Cas9 and TALEN to edit the DMD genetic mutation in canine Duchenne muscular dystrophy
Source: PLoS One. 2020 Jan 21;15(1):e0228072. doi: 10.1371/journal.pone.0228072 (PMC6974172; doi:10.1371/journal.pone.0228072)
Supplement: S8 Table — (DOCX) [file pone.0228072.s020.docx]

|  | **Miercoles** | **Friendly** | **Bubbles** | **Clove** | **Gantu** | **Hera** |
| --- | --- | --- | --- | --- | --- | --- |
| **HDR-tx** | 2.149 | 2.331 | 2.819 | 5.148 | 3.371 | 5.329 |
| **Saline** | 2.101 | 2.466 | 2.761 | 5.445 | 3.295 | 5.219 |
