# Supplementary figures and images for: Correction: Challenges associated with homologous directed repair using CRISPR-Cas9 and TALEN to edit the DMD genetic mutation in canine Duchenne muscular dystrophy
Source: PLoS One. 2020 Oct 22;15(10):e0241430. doi: 10.1371/journal.pone.0241430 (PMC7580940; doi:10.1371/journal.pone.0241430)

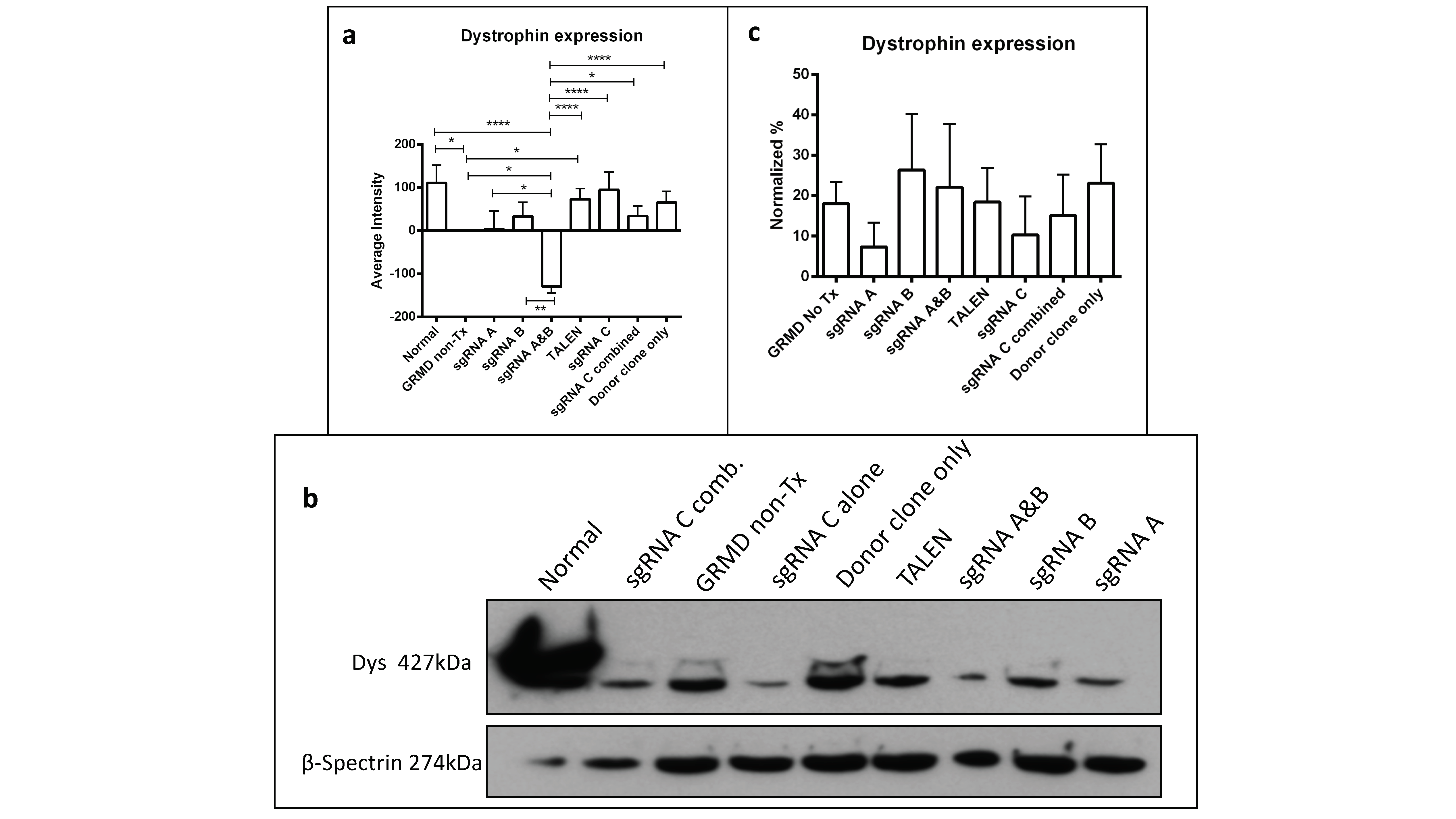

Supplement: S4 Fig — (a) Immunofluorescence microscopy: GRMD non-Tx cells had lower dystrophin expression compared to normal. Levels in sgRNA A-Tx, sgRNA B-Tx, sgRNA C, sgRNA C combined and donor clone only treated cells did not differ from normal, suggesting a potential treatment effect. However, this was not significantly different from non-Tx GRMD or normal cells. TALEN-Tx cells showed an increase in dystrophin protein when compared to non-Tx GRMD cells. Dystrophin expression for the two guides combined was significantly reduced compared to GRMD non-Tx and normal control. Intensity of dystrophin signal from multinucleated myotubes measured with ImageJ and analyzed via one way ANOVA. **** p ≤ 0.0001; * p ≤ 0.05. (b) Western blot: Dystrophin and β-spectrin signal for different treatments. β -spectrin was used as a loading control. (c) Western blot: Quantification of dystrophin signal normalized to normal myotubes protein extract. One way ANOVA was used and no statistical differences were found between treatments. Vertical bars indicate standard error of the mean. (TIFF) [file pone.0241430.s001.tiff]

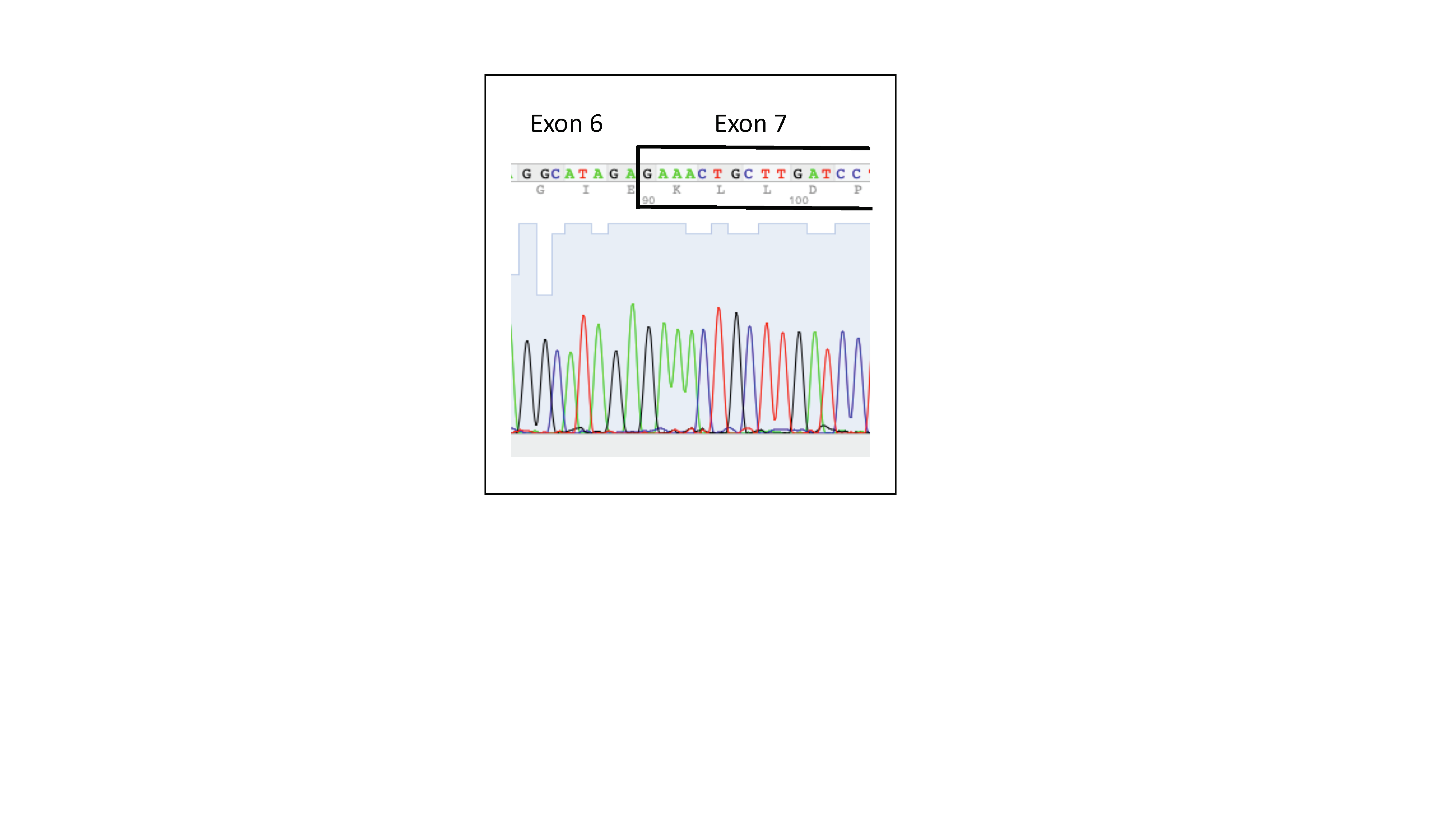

Supplement: S5 Fig — Exon 7 boundary area sequenced from HDR-CRISPR-Tx muscle. Exon 7 was included in the DMD mRNA of the gene edited muscle. (TIFF) [file pone.0241430.s002.tiff]

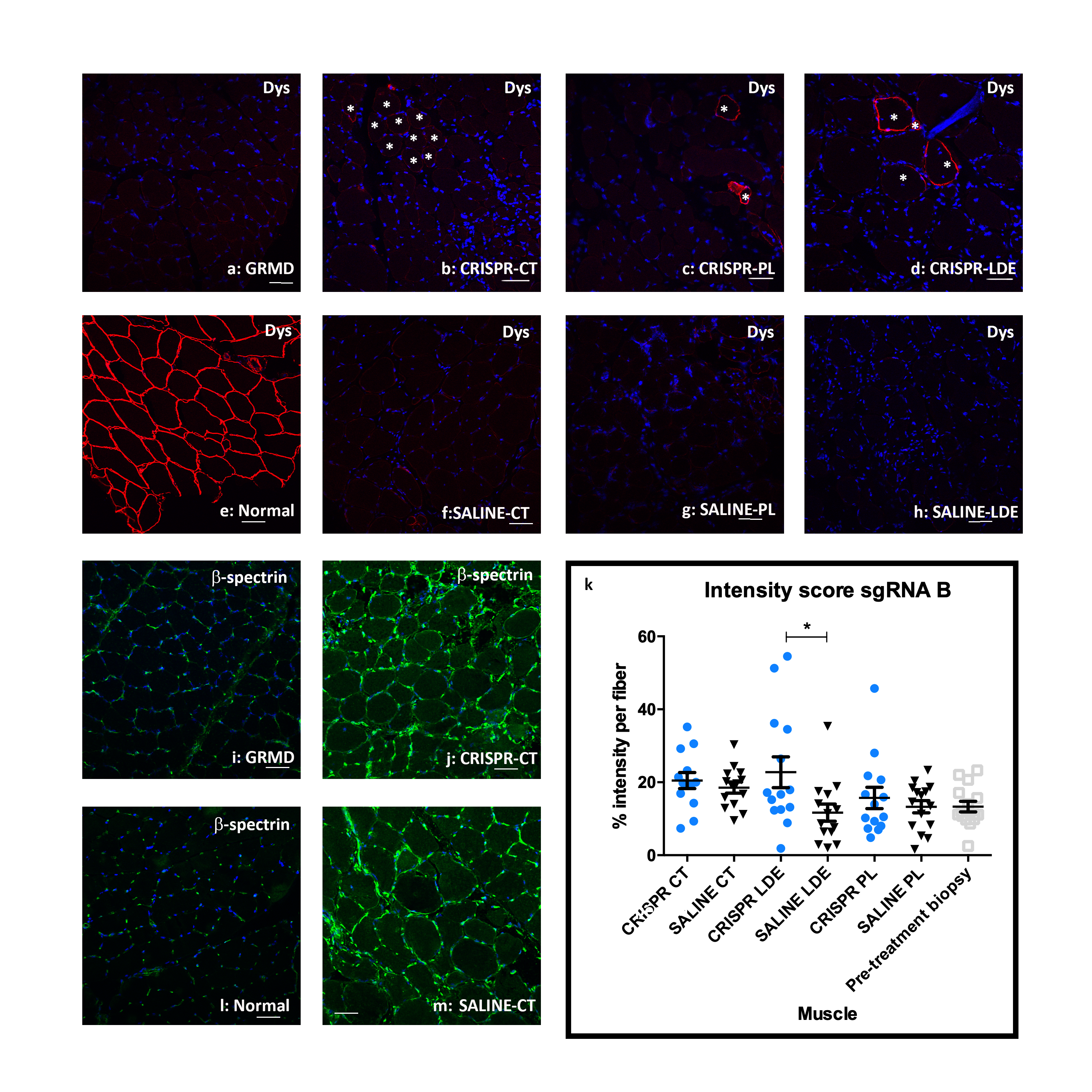

Supplement: S8 Fig — Dystrophin co-stained with C and N-terminus antibodies with Alexa 647 (red), β -spectrin membrane control (green) and DAPI denotes the nuclei (blue). Asterisks denotes cells with a value of 2 in intensity score for dystrophin signal in the GRMD non-Tx and Tx samples. Scale bar = 50μm. (a) Pre-treatment biopsy sample for Bubbles (b) HDR-CRISPR injected cranial tibial (CT) Bubbles (c) HDR-CRISPR injected peroneus longus (PL) Bubbles (d) HDR-CRISPR injected long digital extensor (LDE) Bubbles (e) normal dog muscle (f) SALINE injected CT Bubbles (g) SALINE injected PL Bubbles (h) SALINE injected LDE Bubbles. (i) Pre-treatment biopsy sample for Bubbles (j) HDR-CRISPR injected CT Bubbles (k) dystrophin intensity quantification for Bubbles and Clove via One-way ANOVA multiple comparisons test, blue circle indicates CRISPR-Tx limb, black triangle indicates Saline-Tx limb, gray square indicates pre-treatment biopsied sample; *p<0.05 (l) normal dog muscle (m) SALINE injected CT Bubbles. Dys = dystrophin. (TIF) [file pone.0241430.s003.tif]

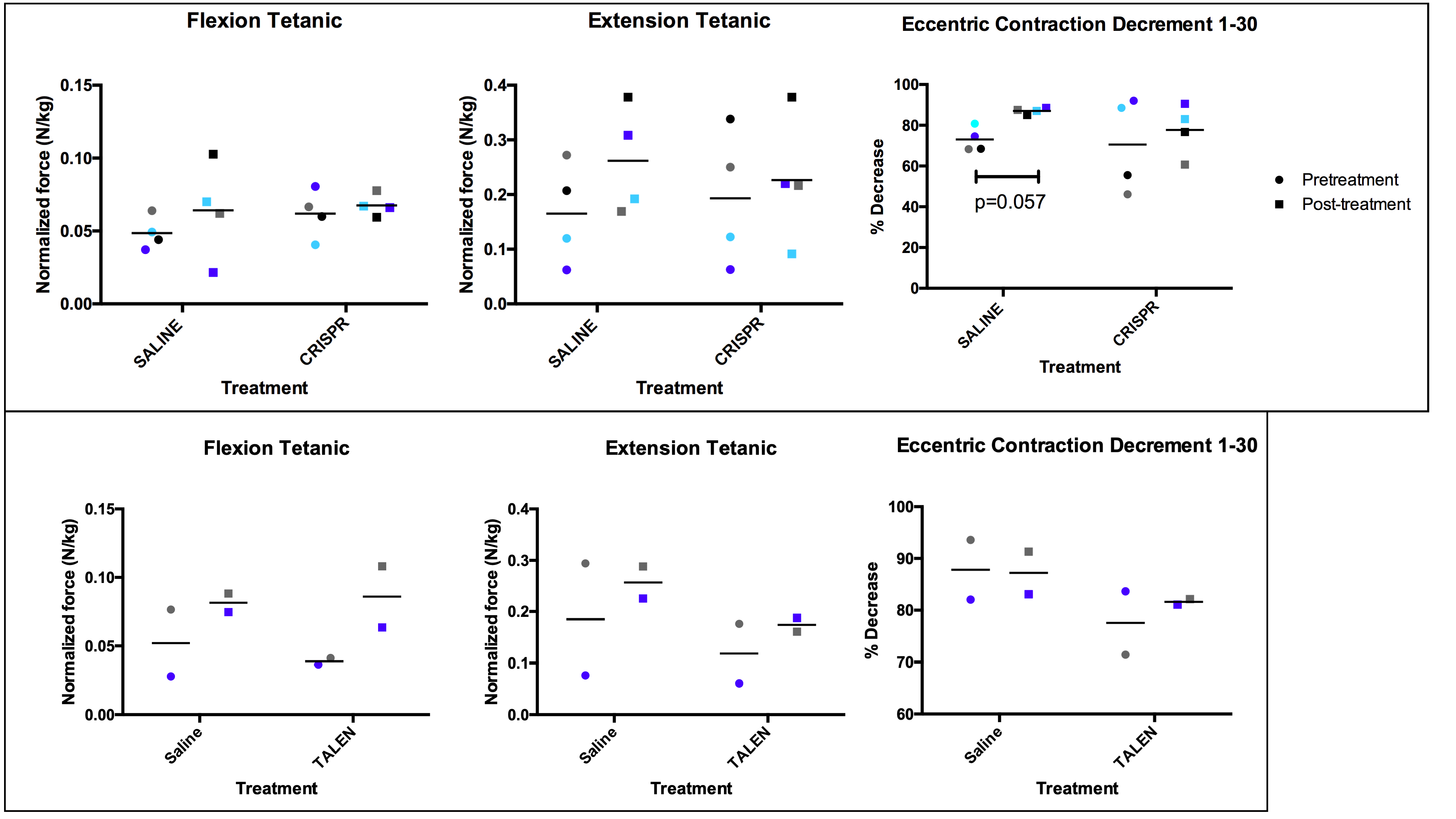

Supplement: S12 Fig — Circle is for pre-treatment values square is for post-treatment. Top: N = 4 analyzed via two way ANOVA. Blue color symbolizes sgRNA B’ data, grey color is for sgRNA A data. Bottom: N = 2 analyzed via two way ANOVA. From left to right: Flexion tetanic values. Extension tetanic values between saline and HDR-injected limbs as well as pretreatment and post-treatment. Eccentric contraction decrement (ECD). No statistical differences were found between saline and HDR-Tx limbs of GRMD dogs. In the saline injected limb for HDR-CRISPR ECD, there was a trend (p = 0.057) for an increase in ECD in post-treatment muscle compared to pre-treatment. The HDR-CRISPR injected limb ECD measurements were similar pre and post-treatment. (TIFF) [file pone.0241430.s004.tiff]
